# RAB33B recruits the ATG16L1 complex to the phagophore via a noncanonical RAB binding protein

**DOI:** 10.1080/15548627.2020.1822629

**Published:** 2020-09-22

**Authors:** Supansa Pantoom, Georgios Konstantinidis, Stephanie Voss, Hongmei Han, Oliver Hofnagel, Zhiyu Li, Yao-Wen Wu

**Affiliations:** a Department of Chemistry, Umeå Centre for Microbial Research, Umeå University, Umeå, Sweden; b Translational Neurodegeneration Section “Albrecht-kossel”, Department of Neurology, University Medical Center Rostock , Rostock, Germany; c Institute of Molecular Biology and Biotechnology,Foundation for Research and Technology-Hellas , Crete, Greece; d Chemical Genomics Centre of the Max Planck Society, Dortmund, Germany; e Max-Planck-Institute of Molecular Physiology, Dortmund, Germany; f National Cancer Center, National Clinical Research Center for Cancer, Cancer Hospital, Chinese Academy of Medical Sciences and Peking Union Medical College, Beijing, China

**Keywords:** ATG12–ATG5-ATG16L1 complex, ATG16L1, autophagosome formation, autophagy, crystal structure, RAB33B, RAB33B-ATG16L1 complex

## Abstract

Autophagosome formation is a fundamental process in macroautophagy/autophagy, a conserved self-eating mechanism in all eukaryotes, which requires the conjugating ATG (autophagy related) protein complex, ATG12–ATG5-ATG16L1 and lipidated MAP1LC3/LC3 (microtubule associated protein 1 light chain 3). How the ATG12–ATG5-ATG16L1 complex is recruited to membranes is not fully understood. Here, we demonstrated that RAB33B plays a key role in recruiting the ATG16L1 complex to phagophores during starvation-induced autophagy. Crystal structures of RAB33B bound to the coiled-coil domain (CCD) of ATG16L1 revealed the recognition mechanism between RAB33B and ATG16L1. ATG16L1 is a novel RAB-binding protein (RBP) that can induce RAB proteins to adopt active conformation without nucleotide exchange. RAB33B and ATG16L1 mutually determined the localization of each other on phagophores. RAB33B-ATG16L1 interaction was required for LC3 lipidation and autophagosome formation. Upon starvation, a fraction of RAB33B translocated from the Golgi to phagophores and recruited the ATG16L1 complex. In this work, we reported a new mechanism for the recruitment of the ATG12–ATG5-ATG16L1 complex to phagophores by RAB33B, which is required for autophagosome formation.

Abbreviations

: ATG: autophagy-related; Cα: alpha carbon; CCD: coiled-coil domain; CLEM: correlative light and electron microscopy; DTE: dithioerythritol; EBSS: Earle’s balanced salt solution; EDTA: ethylenediaminetetraacetic acid; EGFP: enhanced green fluorescent protein; FBS: fetal bovine serum; FLIM: fluorescence lifetime imaging microscopy; FRET: Förster resonance energy transfer; GDP: guanosine diphosphate; GOLGA2/GM130: golgin A2; GppNHp: guanosine 5ʹ-[β,γ-imido]triphosphate; GST: glutathione S-transferase; GTP: guanosine triphosphate; GTPγS: guanosine 5ʹ-O-[gamma-thio]triphosphate; HA (tag): hemagglutinin (tag); HEK: human embryonic kidney; HeLa: Henrietta Lacks; HEPES: (4-(2-hydroxyethyl)-1-piperazineethanesulfonic acid); IgG: immunoglobulin G; K_d_: dissociation constant; MAP1LC3/LC3: microtubule associated protein 1 light chain 3; MCF7: Michigan cancer foundation-7; MEF: mouse embryonic fibroblast; MEM: minimum essential medium Eagle; MST: microscale thermophoresis; NEAA: non-essential amino acids; PBS: phosphate-buffered saline; PE: phosphatidylethanolamine; PtdIns3P: phosphatidylinositol-3-phosphate; RAB: RAS-associated binding; RB1CC1/FIP200: RB1 inducible coiled-coil protein 1; RBP: RAB-binding protein; SD: standard deviation; SDS: sodium dodecyl sulfate; SQSTM1/p62: sequestosome 1; TBS-T: tris-buffered saline-tween 20; WD (repeat): tryptophan-aspartic acid (repeat); WIPI2B: WD repeat domain phosphoinositide interacting 2B; WT: wild type

## Introduction

Autophagy is an evolutionarily conserved catabolic process mainly to recycle or eliminate dysfunctional cellular organelles or proteins. The process involves the formation of double-membrane vesicle structures, termed autophagosomes, which sequester and engulf cytoplasmic components constitutively, or upon nutrient deprivation or stress. The subsequent autophagosome maturation is achieved by fusing with lysosomes to form autolysosomes, leading to the exposure of the cargo to lysosomal hydrolases for digestion. Autophagy plays an important role in cellular physiology including cell development and has been associated with diverse human diseases, including cancer, neurodegeneration and pathogen infection [1–3].

In mammalian cells, autophagosomes initiate from phagophores throughout the cytosol. Phagophores expand, enfold cytosolic cargo and finally close, forming autophagosomes. Two ubiquitin-like conjugation systems are crucial for the elongation and closure of autophagosomes [4,5]. Atg8 (in yeast) and its mammalian homologs MAP1LC3/LC3 (microtubule associated protein 1 light chain 3), GABARAP (GABA type A receptor-associated protein) and GABARAPL2/GATE-16 (GABA type A receptor associated protein like 2) are conjugated to phosphatidylethanolamine (PE) lipid in a reaction controlled by the ATG12–ATG5-ATG16L1 complex. Lipidated Atg8/LC3 is essential for autophagosome biogenesis. Atg8–PE/LC3–PE can promote membrane tethering and hemifusion [6–8]. The ATG12–ATG5 conjugate forms a complex with ATG16L1, which exhibits E3-like activity to facilitate the conjugation of LC3 to PE. However, the molecular mechanism by which the ATG12–ATG5-ATG16L1 complex is targeted to phagophores remains largely unclear. RB1CC1/FIP200 (RB1 inducible coiled-coil protein 1) and the phosphatidylinositol-3-phosphate (PtdIns3P) binding protein, WIPI2B (WD repeat domain, phosphoinositide interacting 2B), have been implicated in the recruitment of the ATG12–ATG5-ATG16L1 complex to the membrane. However, the binding of WIPI2B to ATG16L1 is rather weak [9–12]. Three amino acid residues at the coiled-coil domain (CCD) of ATG16L1 are involved in direct binding to phosphoinositides on the membrane [13]. The tryptophan-aspartic acid (WD) repeat domain and the C-terminal membrane-binding region of ATG16L1 are required for the recruitment of ATG16L1 to single membranes during non-canonical autophagy [14,15].

RAS-associated binding (RAB) proteins are key regulators of intracellular vesicle transport in eukaryotic cells [16,17]. RAB proteins are post-translationally modified at the C terminus with prenyl groups that function as membrane anchors. RAB proteins switch between an active guanosine triphosphate (GTP)-bound and inactive guanosine diphosphate (GDP)-bound state. In their GTP-bound form, RAB proteins orchestrate vesicular transport by recruiting functionally diverse effectors including tethering complexes and motor proteins. A few RAB proteins have been implicated in autophagosome formation and maturation [18,19], including RAB7 [20,21], RAB5 [22–24], RAB11 [25,26] and RAB1 [27–30]. Golgi-resident RAB33B interacts with ATG16L1 [31]. Its GTPase-activating protein (GAP), TBC1D25/OATL1 (TBC1 domain family member 25), negatively regulates autophagosome maturation by deactivating RAB33B [32]. However, the mechanisms of action of RAB proteins in autophagosome formation remain elusive. It is important to understand how RAB proteins function distinctively in vesicular transport and autophagosome formation. Although many of RAB-effector complexes involved in vesicular transport have been elucidated [33], the molecular basis for RAB recognition by autophagy-specific proteins is not clear. Here, we combined structural and cell biological approaches to address how the ATG16L1 complex is recruited to the membrane. Our work revealed that ATG16L1 is a non-canonical RAB binding protein of RAB33B and provided insights into the molecular mechanism of their binding. We demonstrated that RAB33B plays a key role in the recruitment of the ATG16L1 complex to the phagophore and is essential for autophagosome formation.

## Results

### Structure and interaction of the RAB33B-ATG16L1 complex

**Figure 1.**
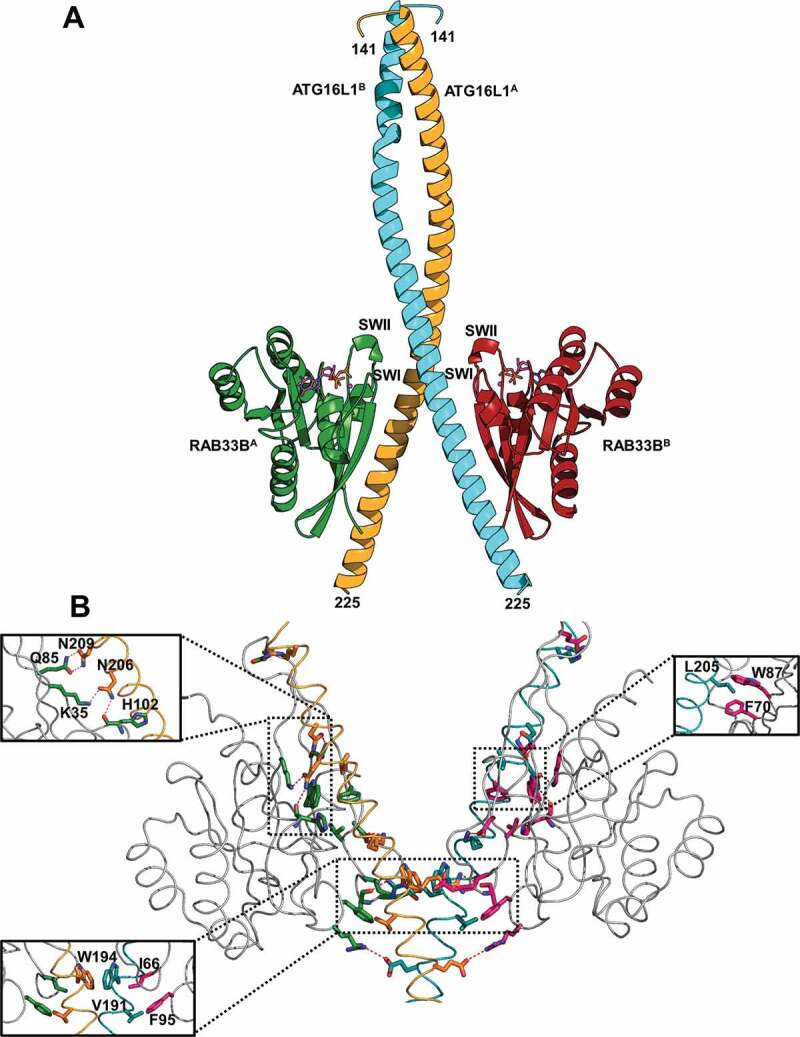
Overall structure and interaction of RAB33B with ATG16L1 CCD. (A) Cartoon representation of GTP-bound RAB33B-ATG16L1 CCD complex. ATG16L1^A^ and ATG16L1^B^ molecules are shown in orange and cyan, respectively. The RAB33B^A^ and RAB33B^B^ molecules are displayed in green and red, respectively. The switch I and switch II regions are labeled as SWI and SWII, respectively. The N- and C-terminal residues of ATG16L1 CCD are indicated (141 and 225, respectively). (B) Detailed view of the RAB33B-ATG16L1 binding interface. The backbone of RAB33B, ATG16L1^A^ and ATG16L1^B^ are colored in gray, orange and cyan, respectively. Interacting residues of RAB33B and ATG16L1 are represented as stick models and red dotted lines indicate hydrogen bonds

**Figure 2.**
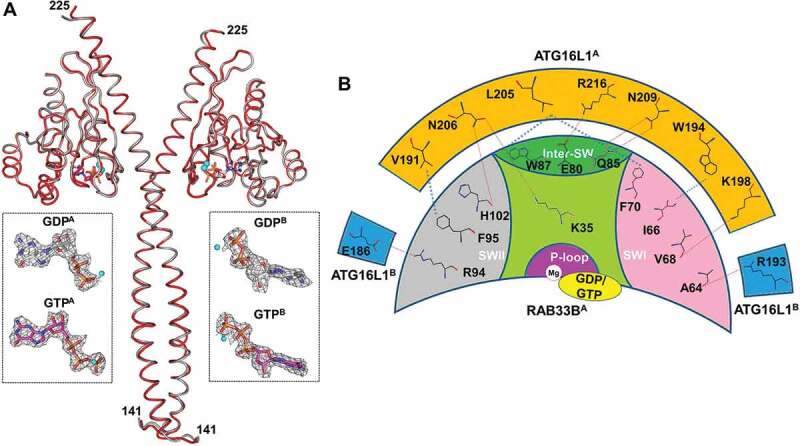
Structures of GTP-RAB33B-ATG16L1 and GDP-RAB33B-ATG16L1 complexes and the ATG16L1-RAB33B binding interface. (A) GTP- and GDP-bound complexes are shown as red and gray Cα backbone, respectively. The GTP and GDP molecules are shown in indexes as stick model with carbon atoms colored in pink and gray, respectively. The 2*F*_o_-*F*_c_ density map contoured with 1.0 σ. (B) RAB33B is represented as a crescent moon, where the switch I (SWI), switch II (SWII), Inter-switch (Inter-SW) and P-loop are indicated in pink, gray, dark green and violate areas, respectively. The orange and blue boxes represent ATG16L1^A^ and ATG16L1^B^, respectively. Hydrogen bonding interactions with a cutoff of 3.5 Å are shown with red dashed lines. The hydrophobic interactions are shown in blue dashed lines

We prepared the ATG16L1(141–265) complexes with GDP-bound RAB33B(30–218) or GTP-bound RAB33B^Q92L^(30–218). Their crystal structures were determined at 2.4 Å. In both structures, GTP- and GDP-bound RAB33B-ATG16L1 complexes adopt the same conformation (Figures 1A and 2A). The complex showed a dyad symmetry with a 2:2 configuration. Two molecules of ATG16L1(141–265) formed a homodimer, within which the dimeric helices of ATG16L1 formed a parallel coiled-coil structure (residues 161–194). The C-terminal segments of two helices (residues 195–225) are detached from each other, where one RAB33B molecule binds independently to each helix. No interaction was observed between the two RAB33B molecules in the crystal structure, suggesting that RAB33B molecules do not cooperate for binding to ATG16L1 (Figure 1B). Each RAB33B bound one molecule of GTP or GDP and one Mg^2+^ with well-defined electron density (Figure 2A). The structures of both RAB molecules were identical with the classical folding of RAB GTPases which contain a central six-strand β-sheet (β1-β6) flanked by five α-helices (α1-α5) (Figure 1A).

The crystal structure of the yeast Atg16 CCD (PDB 3A7P) shows a similar parallel coiled-coil structure from residues 55 to 142 which correspond to residues 118 to 205 of ATG16L1 (Figure S1) [34]. However, only part of the coiled-coil structure (residues 161 to 194) was observed in ATG16L1, whereas the coiled-coil structure of residues 141–160 was distorted and *a-a*´ and *d-d*´ pairs were not observed. The distorted coiled-coil structure might be caused by the absence of N-terminal residues of the ATG16L1 CCD which play a role in stabilizing the coiled-coil structure (Figure S2A).

The coiled-coil region of ATG16L1 was packed into a typical parallel coiled-coil structure with residues at position *a* and *d* of the heptad repeat form *a-a*´ and *d-d*´ packing interface to stabilize the coiled coil structure. All residues at the *d* position were hydrophobic residues, with mostly leucine residues. These leucine pairs formed leucine zippers to stabilize the dimer structure, whereas in the position *a*, bulky hydrophobic residues, tryptophan, phenylalanine and tyrosine were formed pairwise. Moreover, the polar side chain of asparagine was also observed at the *a* position (Figure S2B).

Notably 40 residues (226–265) at the C terminus did not show any defined electron density, suggesting a flexible region. 3D structure modeling and secondary structure prediction suggested that the region (residues 225 to 326) is probably non-structured, which could represent a long loop connecting the CCD with the WD repeat domain (Figure S1).

RAB33B interacted with ATG16L1 mainly through its switch I, switch II and inter switch regions, whereas both the CCD and the C-terminal detached helices of ATG16L1 homodimer were involved in the binding with RAB33B (Figure 1A, 2B and S3A). The core hydrophobic interaction formed by invariant, hydrophobic dyad residues of I66 and F95 in the switch regions of RAB33B^A^ molecule (molecule A of the dimeric complex) with W194 and V191 in the CCD of ATG16L1^A^ molecule, respectively. There was a hydrophobic interaction between I66 and F95 of RAB33B. Moreover, W194 of ATG16L1 was involved in *a-a’* hydrophobic pair in the CCD, thus, a hydrophobic interaction cluster formed between two RAB33B molecules and both helices of ATG16L1 CCD (Figure 1B). In addition, cross interactions between RAB33B^A^ and ATG16L1^B^ molecules were also observed. R94 of RAB33B^A^ formed a salt bridge with E186 of ATG16L1^B^, while the main chain carbonyl group of A64 of RAB33B^A^ was hydrogen-bonded with R193 of ATG16L1^B^ (Figure 2B). These suggested that RAB33B may stabilize a dimeric ATG16L1 structure. In contrast to the CCD, C-terminal detached helix of ATG16L1 was involved in binding with an individual RAB33B molecule (Figures 1B and 2B). L205 of ATG16L1 made hydrophobic contacts with F70 and W87, two of invariant, hydrophobic triad residues of RABs (F70, W87 and Y103), which have been proposed to be important for RAB-effector interactions (Figure 1B and S3B) [35]. Two key hydrogen bonding interactions included interaction of N206 of ATG16L1 with K35 and the main chain carbonyl group of H102 of RAB33B and hydrogen bonds between N209 of ATG16L1 and Q85 of RAB33B (Figures 1B and 2B).

### The GDP-bound RAB33B adopts the active conformation upon ATG16L1 binding

**Figure 3.**
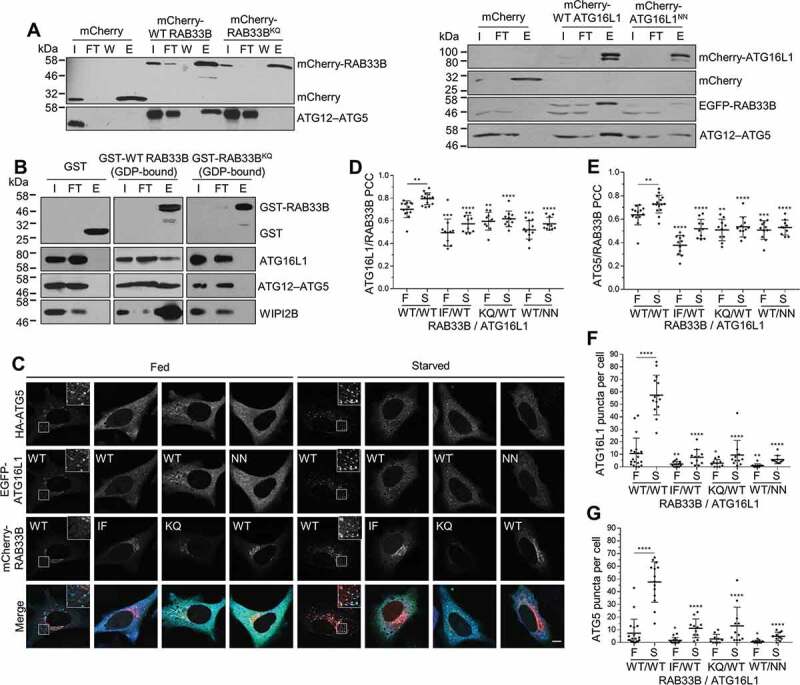
Interaction and localization of ATG16L1 and RAB33B in cells. (A) HEK293T cells transiently expressing mCherry or the indicated mCherry-RAB33B (left) or mCherry-ATG16L1 (right) constructs were incubated in EBSS for 2 h, lysed and mCherry-tagged proteins were immunoprecipitated using the RFP-trap and subjected to immunoblot analysis. I: Input, FT: Flow-through, W: Wash, E: Elution. (B) Recombinant wild-type or mutant GST-RAB33B were incubated with cell lysate from HeLa cells previously incubated in EBSS for 2 h as indicated. Samples were subjected to affinity isolation using glutathione beads and immunoblot analysis. WIPI2B binding was trapped by crosslinking. (C) HeLa cells transiently expressing HA-ATG5 and the indicated EGFP-ATG16L1 and mCherry-RAB33B constructs were cultured for 48 h. Cells were incubated in complete medium (Fed) or EBSS (Starved) for 2 h. Subsequently, cells were fixed, subjected to immunostaining for HA and imaged under confocal microscopy. Inset shows magnification of the boxed region. Scale bar: 10 μm. (D, E) Pearson’s colocalization coefficient between EGFP-ATG16L1 (D) or HA-ATG5 (E) and mCherry-RAB33B from (C). Data are represented as mean ± SD, n = 10–15 cells. (F, G) Quantification of EGFP-ATG16L1 (F) or HA-ATG5 (G) puncta per cell from (C). Data are represented as mean ± SD, n = 10–19 cells. The statistical analysis for mutants shown in (D-G) represent comparison to the respective (F or S) WT/WT condition. WT: wild type, IF: RAB33B^I66A,F95A^, KQ: RAB33B^K35A,Q85A^, NN: ATG16L1^N206A,N209A^, F: Fed, S: Starved. *p ≤ 0.05, **p ≤ 0.01, ***p ≤ 0.001, ****p ≤ 0.0001 (Student’s t-Test)

To our surprise, both GDP- and GTP-bound RAB33B adopted the same conformation in the complexes. To understand this observation, we determined the binding affinity of ATG16L1 to guanosine 5ʹ-[β,γ-imido]triphosphate (GppNHp, a non-hydrolysable GTP analog)- or GDP-bound RAB33B by microscale thermophoresis (MST). The MST measurements showed that both forms of RAB33B strongly bound to ATG16L1 with dissociation constant (K_d_) of 95 nM for GppNHp-RAB33B, and K_d_ of 213 nM for GDP-RAB33B with GppNHp-bound RAB33B binding only approximately 2-fold higher than GDP-bound RAB33B (Table 1 and Figure S4). Independent measurements using isothermal titration calorimetry (ITC) also show that ATG16L1 binds to GDP- and guanosine 5ʹ-O-[gamma-thio]triphosphate (GTPγS)-RAB33B in a similar affinity [36]. Moreover, GDP-bound wild type (WT) RAB33B could pull down the endogenous ATG16L1 complex from cells (Figure 3B). These results indicated that ATG16L1 binding is independent of the RAB33B nucleotide-binding state. Since effectors by definition only bind the GTP-bound form of RAB, ATG16L1 is essentially not a canonical RAB effector but rather a RAB binding protein (RBP). Structural comparison of GTP- and GDP-bound RAB33B molecules in complexes with ATG16L1 revealed that their conformations are identical with an alpha-carbon root-mean square deviation (Cα-RMSD) of 0.16 Å. GTP- and GDP-bound RAB33B form similar interface with ATG16L1 with surface areas of 847 Å^2^ and 833 Å^2^, respectively. Moreover, the conformations of both complexed RAB33B molecules were very similar to the non-complexed GppNHp-bound RAB33B (PDB 1Z06) [37] with an alpha-carbon root-mean square deviation of 0.38 Å for GTP-bound form and 0.40 Å for GDP-bound form, suggesting that ATG16L1 binding does not induce significant conformational change of active RAB33B (Figure S3B). The side chains of I66 and F95 dyad residues adopted the same configurations in all three structures. However, the configurations of the side chains of F70, W87 and Y103 triad residues in complexed RAB33B molecules were different from those in the non-complexed GppNHp-bound RAB33B (Figure S3B), suggesting structural plasticity of these residues [35].

**Table 1.** Dissociation constants for interactions between RAB33B and ATG16L1

RAB33B|ATG16L1(141–265)	K_d_ (µM)	Fold change
WT^GppNHp^|WT	0.095 ± 0.013	1
WT^GDP^|WT	0.213 ± 0.045	2.2
WT^GppNHp^|N206A	3.383 ± 0.286	36
WT^GppNHp^|N209A	0.884 ± 0.126	9
WT^GppNHp^|N206A,N209A	24.133 ± 0.586	254
I66A^GppNHp^|WT	25.900 ± 2.862	273
F95A^GppNHp^|WT	17.050 ± 2.267	179
I66A,F95A^GppNHp^|WT	Not detectable	
K35A^GppNHp^ |WT	>100	>1000
Q85A^GppNHp^ |WT	Not detectable	

Data are represented as mean ± SD, n = 3 measurements.

The conformations of GTP- and GDP-bound RAB33B complexes remained identical at the switch I and switch II regions, where the major conformational changes are typically observed in different nucleotide binding states. To investigate the conformational change between the GDP- and GTP-bound RAB33B, the GDP-bound RAB1A (PDB 2FOL) with 44% sequence identity to RAB33B was chosen as a model. ATG16L1 binding induced a remarkable conformational change of the switch I region. This results in a ca. 8.4 Å translocation of the Cα atom of I66, followed by a hydrophobic packing of I66 against F95 of RAB33B and W194 of ATG16L1 (Figure S3C). The switch II region is largely disordered in GDP-bound RAB1A, while the switch II region of RAB33B adopted the active conformation and formed extensive interactions with ATG16L1. Therefore, ATG16L1 induced conformational changes of GDP-bound RAB33B to hold it in the active conformation.

### Biochemical characterization of the RAB33B-ATG16L1 interaction

Mutation of N206 or N209 to alanine (N206A or N209A) in ATG16L1 reduced the binding affinity by 36-fold and 9-fold, respectively. Double mutation (N206A, N209A) dramatically reduced the binding affinity by 254-fold. Consistently, mutation of the N206 and N209 binding residues, K35 or Q85 in RAB33B to alanine (K35A or Q85A) abolished the binding of RAB33B to ATG16L1. Mutation of I66 or F95 in RAB33B to alanine (I66A or F95A) led to reduction of binding affinity by 273-fold and 179-fold, respectively, while mutation of both residues (I66A, F95A) completely disrupted the binding of RAB33B to ATG16L1 (Table 1 and Figure S4). These interactions were further confirmed in cells by affinity-isolation experiments (Figure 3A and 3B). To confirm the function of RAB33B mutants, we showed that the RAB33B mutants display similar *in vitro* prenylation activity to the wild type protein, suggesting proper binding to CHM/REP-1 (CHM Rab escort protein) and RABGGT (Rab geranylgeranyltransferase) (Figure S3D) [38,39]. Moreover, ATG16L1^N206A,N209A^ (NN mutant) bound to the endogenous ATG12–ATG5 complex but not to RAB33B (Figure 3A). These measurements suggested that the hydrophobic interaction of I66 and F95 and the hydrogen bonding interaction of K35 and Q85 play an important role in binding and recognition of RAB33B to ATG16L1.

Alignment of the RAB33B sequence with other RAB sequences revealed that most of the residues involved in the RAB33B-ATG16L1 interaction are conserved in the RAB protein family (Figure S3A). In order to understand how RAB33B specifically recognizes the autophagic RBP ATG16L1, we compared our structure to the structures of other RAB-effector complexes with coiled-coil structures, e.g. RAB5-RABEP1/rabaptin-5 (rabaptin, RAB GTPase binding effector protein 1) (PDB 1TU3), RAB11-OPTN/FIP2 (optineurin) (PDB 2GZD), RAB7-RILP (RAB interacting lysosomal protein) (PDB 1YHN) and RAB6-GCC2/GCC185 (GRIP and coiled-coil domain containing 2) (PDB 3BBP). Similar to the RAB33B-ATG16L1 complex, the overall structures of these complexes are organized in a dyad symmetric complex. A conserved hydrophobic interaction mediated by a dyad of invariant hydrophobic residues of RABs was identified. Isoleucine in the TIGVDF motif (RAB33B I66, RAB5 I53, RAB7 I41, RAB6 I46 and RAB11 I44) in the switch I region makes hydrophobic interactions with phenylalanine or tyrosine in the ERF motif (RAB33B F95, RAB5 Y82, RAB7 F70 and RAB6 F75, except for RAB11 W73) in the switch II region. These two residues formed direct hydrophobic interactions and/or van der Waals contacts with residues in RAB effectors (Figure S3E).

In other RAB-effector complexes, e.g. RAB3A-RPH3A (rabphilin 3A) (PDB 1ZBD), RAB4-RBSN/Rabenosyn-5 (rabenosyn, RAB effector) (PDB, 1Z0K), RAB5A-EEA1 (early endosome antigen 1) (PDB 3MJH) and RAB6-DENND5A/RAB6IP1 (DENN domain containing 5A) (PDB 3CWZ), which are not arranged in a dyad symmetry, there are also such conserved interactions mediated by the invariant hydrophobic dyad residues of RABs (Figure S3E). These findings suggested that the invariant hydrophobic dyad residues of RABs, i.e. isoleucine in the TIGVDF motif and phenylalanine in the ERF motif, play a critical role in RAB recognition of effectors.

Besides the conserved hydrophobic interactions, we found a new binding interface in the RAB33B-ATG16L1 complex, which was not observed in other RAB-effector complexes (Figure S3E). Such an interface comprised hydrogen bond interactions of K35 and Q85 of RAB33B with N206 and N209 of ATG16L1 (Figure 1B), respectively. As shown by mutagenesis analysis, the hydrogen bond interactions are essential for RAB33B-ATG16L1 binding and might determine the selectivity for RAB33B recognition of the autophagic RBP, ATG16L1.

### RAB33B interaction with ATG16L1 is essential for their phagophore localization

Previous studies in our lab and others demonstrate that RAB effectors play an important role in subcellular localization of RAB proteins [38,40]. We studied the role of RAB33B-ATG16L1 binding in their membrane localization. The ATG12–ATG5-ATG16L1 complex predominantly localizes to the phagophore, and ATG16L1 may also localize to autophagosome precursors [41,42]. To investigate the localization of RAB33B on the phagophore, we co-expressed RAB33B with ATG16L1 and ATG5. Upon starvation, enhanced colocalization between WT RAB33B and WT ATG16L1 (80 ± 5%) or ATG5 (73 ± 8%) were observed. In contrast, RAB33B^I66A,F95A^ (IF mutant), RAB33B^K35A,Q85A^ (KQ mutant) and ATG16L1^N206A,N209A^ (NN mutant) showed approximately 20% reduction in colocalization under fed and starved conditions (Figure 3C-E). Moreover, in cells expressing RAB33B mutants (IF or KQ) significantly less ATG16L1-positive and ATG5-positive structures were observed than in the cells expressing WT RAB33B under both fed and starved conditions, with ATG16L1 and ATG5 being predominantly cytosolic. Correspondingly, in cells expressing ATG16L1^NN^, both ATG16L1^NN^ and ATG5 showed mainly cytosolic localization and less puncta formation than in the WT ATG16L1-expressing cells under both fed and starved conditions (Figure 3C, 3F and 3G). To exclude side effects of transient expression, we utilized cells stably expressing EGFP (enhanced green fluorescent protein)-ATG16L1 that appears to show a similar response to autophagic stimulation as endogenous ATG16L1 [43]. Michigan cancer foundation-7 (MCF7) cells stably expressing EGFP-ATG16L1 showed similar change in colocalization with RAB33B or its mutant under starvation, compared to the above results of transiently transfected cells (Figure S5A).

**Figure 4.**
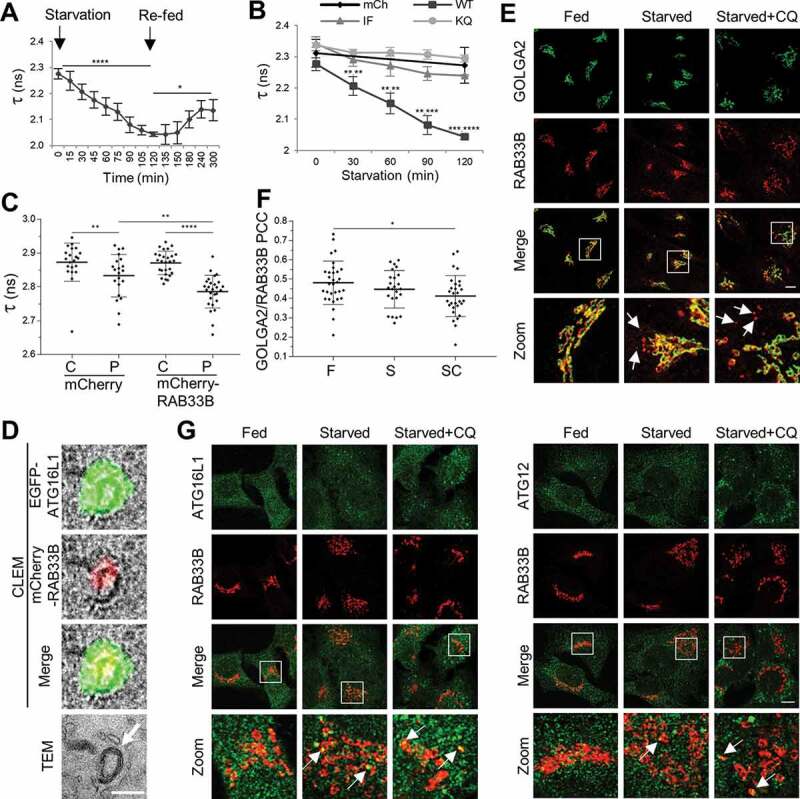
RAB33B interacts with ATG16L1 and translocates to the phagophore upon starvation. (A) Measurement of RAB33B-ATG16L1 interaction in live cells by FLIM. Formation and dissociation of RAB33B-ATG16L1 complex under starved and re-fed conditions. HeLa cells transiently co-expressing mCherry-RAB33B WT and EGFP-ATG16L1 WT were subjected to starvation and complete medium replenishment and time lapse FLIM measurement of EGFP lifetime. Data are represented as mean ± SD, n = 4 cells. (B) HeLa cells transiently co-expressing mCherry or the indicated mCherry-RAB33B and EGFP-ATG16L1 constructs were subjected to EBSS starvation and time lapse FLIM measurement of EGFP lifetime. Data are represented as mean ± SD, n = 4–5 cells. The statistical analysis represents comparison of each WT with the respective IF (left asterisks) or KQ (right asterisks) mutant. (C) RAB33B-ATG16L1 complex formation on punctate structures. HeLa cells transiently co-expressing mCherry-RAB33B WT and mCitrine-ATG16L1 WT were subjected to EBSS starvation for 2 h. Citrine fluorescence lifetime of cytosolic fractions and punctate structures were measured. Data are represented as mean ± SD, of 3 cytosolic (C) and 3 punctate (P) fractions per cell. n = 7–9 cells. (D) CLEM of transiently co-expressed EGFP-ATG16L1 WT and mCherry-RAB33B WT in HeLa cells. A double ATG16L1- and RAB33B-positive structure is depicted. Arrow indicates phagophore-like structure. Scale bar: 0.5 μm. (E) HeLa cells were incubated in complete medium (Fed), EBSS (Starved) or EBSS with 50 μM chloroquine (Starved+CQ) for 2 h. Subsequently, cells were fixed, subjected to immunostaining for endogenous RAB33B and GOLGA2 and imaged under confocal microscope. Magnification of boxed regions is shown at the bottom row. Arrows indicate GOLGA2-negative/RAB33B-positive punctate structures. Scale bar: 10 μm. (F) Pearson’s colocalization coefficient between endogenous RAB33B and GOLGA2 from (E). Data are represented as mean ± SD, n = 24–31 cells. (G) HeLa cells were incubated in complete medium (Fed), EBSS (Starved) or EBSS with 50 μM chloroquine (Starved+CQ) for 2 h. Subsequently, cells were fixed, subjected to immunostaining for endogenous RAB33B and ATG16L1 or ATG12 and imaged under confocal microscopy. Magnification of boxed regions is shown at the bottom row. Arrows indicate colocalization between endogenous RAB33B and ATG16L1 or ATG12. Scale bar: 10 μm. mCh: mCherry, WT: wild type, IF: RAB33B^I66A,F95A^, KQ: RAB33B^K35A,Q85A^, TEM: Transmission Electron Microscopy, F: Fed, S: Starved, SC: Starved+Chloroquine, CQ: Chloroquine. *p ≤ 0.05, **p ≤ 0.01, ***p ≤ 0.001, ****p ≤ 0.0001 (Student’s t-Test)

In order to examine the interaction of RAB33B with ATG16L1 in cells, Förster resonance energy transfer (FRET) between EGFP-ATG16L1 and mCherry-RAB33B was measured in live cells via fluorescence lifetime imaging microscopy (FLIM). If FRET occurs, energy transfer from the donor molecule to the acceptor molecule will cause a decrease in the lifetime of the donor, which can be measured by FLIM. Since FLIM-FRET measurements are insensitive to the concentrations of the fluorophores, artifacts caused by changes in concentration and emission intensity can be avoided. Time-dependent decrease of fluorescence lifetime (τ) of EGFP was observed in the cells expressing wild type RAB33B and ATG16L1 upon starvation and the fluorescence lifetime recovered after re-feeding of the cells, suggesting formation of RAB33B-ATG16L1 complexes upon induction of autophagy (Figure 4A). In contrast, cells expressing RAB33B^IF^ or RAB33B^KQ^ showed significant higher average lifetime of EGFP than the WT RAB33-expressing cells after starvation, suggesting less complex formation of RAB33B mutants with ATG16L1 in cells (Figure 4B). Moreover, the fluorescence lifetime of ATG16L1- and RAB33B-positive structures is significantly lower compared to that of the cytosolic ATG16L1, suggesting complex formation on these punctate structures (Figure 4C). Correlative light and electron microscopy (CLEM) data suggested that RAB33B and ATG16L1 colocalize on phagophore-like structures (Figure 4D). Under starved conditions, live-cell imaging of mTurquoise-ATG16L1, EGFP-ATG5 and mCherry-RAB33B suggested that the three proteins initially coincide on a tubular structure and colocalize during the whole life span of the phagophore (Figure S5B and Movie S1).

Endogenous RAB33B largely localized on the Golgi apparatus. Under starved and starved+chloroquine conditions, endogenous RAB33B structures became more dispersed and part of them were negative for the Golgi marker, GOLGA2/GM130 (golgin A2), causing drop of colocalization between RAB33B and GOLGA2 (Figure 4E and 4F). Some endogenous ATG16L1 and ATG12 punctate structures were positive for endogenous RAB33B under starvation. Additionally, many endogenous ATG16L1 and ATG12 puncta colocalized or were in close proximity with the RAB33B-positive structures (Figure 4G). Moreover, the endogenous ATG12–ATG5-ATG16L1 complex co-immunoprecipitated with RAB33B (Figure 3A and 3B). Taken together, part of RAB33B translocated from the Golgi to phagophores when autophagy was induced. Such a translocation was driven by the interaction between RAB33B and ATG16L1, which is important for the biogenesis of phagophores. The results suggested that RAB33B and ATG16L1 mutually determine the localization of each other on the phagophore through formation of a stable complex.

### RAB33B binding to ATG16L1 is required for LC3 lipidation and autophagosome formation

**Figure 5.**
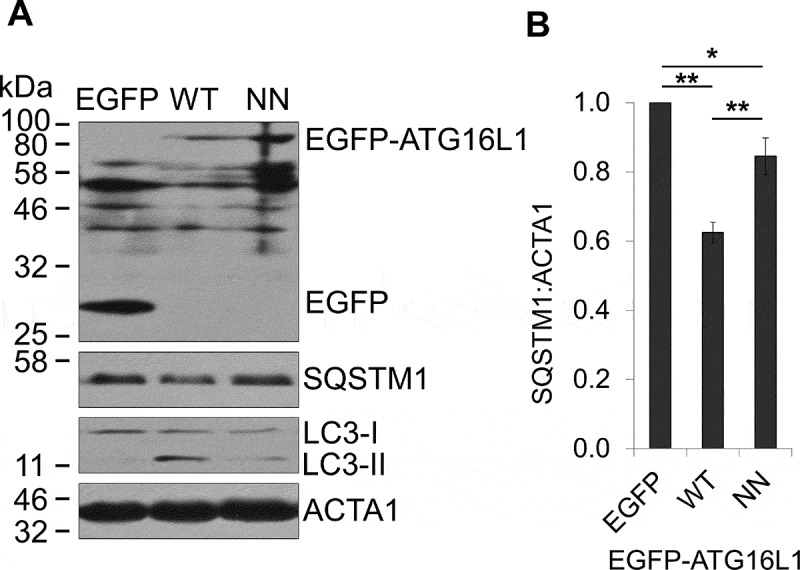
RAB33B-ATG16L1 interaction is essential for autophagy. (A) *atg16l1^−/−^* MEF transiently expressing EGFP or the indicated EGFP-ATG16L1 constructs were incubated in EBSS for 2 h, lysed and subjected to immunoblot analysis. (B) Normalized ratio of SQSTM1:ACTA1 from immunoblots in (A). Data are represented as mean ± SD, from 3 independent experiments. WT: wild type, NN: ATG16L1^N206A,N209A^. *p ≤ 0.05, **p ≤ 0.01 (Student’s t-Test)

**Figure 6.**
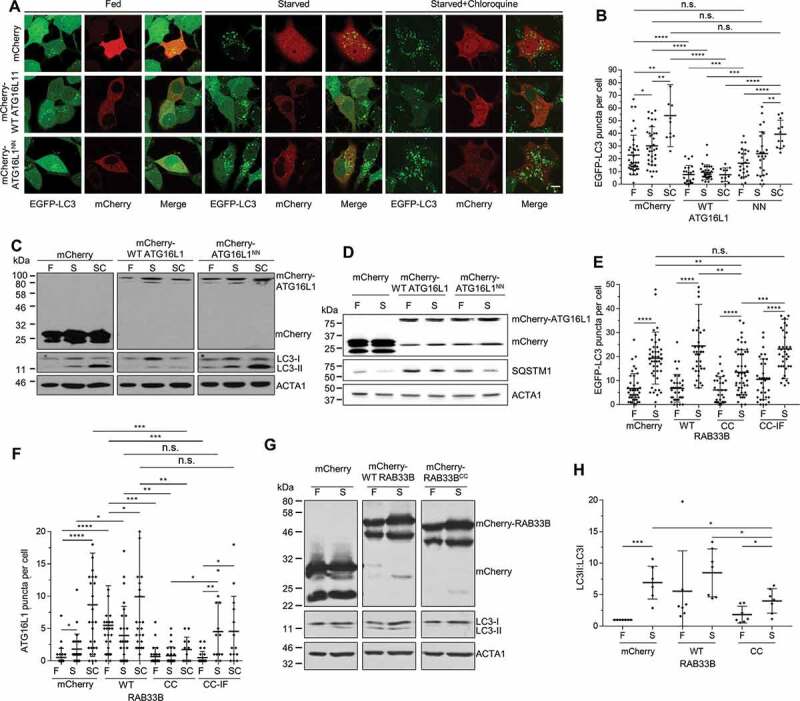
RAB33B-ATG16L1 interaction is essential for autophagosome formation. (A) Stable EGFP-LC3 MCF7 cells transiently expressing mCherry or the indicated mCherry-ATG16L1 constructs were incubated in complete medium (Fed), EBSS (Starved) or EBSS with 50 μM chloroquine (Starved+Chloroquine) for 2 h and imaged by confocal microscopy. Scale bar: 10 μm. (B) Quantification of EGFP-LC3 puncta per cell from (A). Data are represented as mean ± SD, n = 11–40 cells. (C) HEK293T cells transiently expressing mCherry or the indicated mCherry-ATG16L1 constructs were incubated in complete medium (F), EBSS (S) or EBSS with 50 μM chloroquine (SC) for 2 h, lysed and subjected to immunoblot analysis. (D) HEK293T cells transiently expressing mCherry or the indicated mCherry-ATG16L1 constructs were incubated in complete medium (F) or EBSS (S) for 2 h, lysed and subjected to immunoblot analysis. (E) Stable EGFP-LC3 MCF7 cells transiently expressing mCherry or the indicated mCherry-RAB33B constructs were cultured for 48 h. Cells were incubated in complete medium (F) or EBSS (S) for 2 h, imaged under confocal microscopy and EGFP-LC3 puncta per cell were quantified. Data are represented as mean ± SD, n = 32–50 cells. (F) HeLa cells transiently expressing mCherry or the indicated mCherry-RAB33B constructs were incubated in complete medium (F), EBSS (S) or EBSS with 50 μM chloroquine (SC) for 2 h. Cells were subjected to immunostaining for endogenous ATG16L1, imaged by confocal microscopy and ATG16L1 puncta per cell were quantified. Data are represented as mean ± SD, n = 12–30 cells. (G) HEK293 cells transiently expressing mCherry or the indicated mCherry-RAB33B constructs were incubated in complete medium (F) or EBSS (S) for 2 h, lysed and subjected to immunoblot analysis. (H) Quantification of immunoblot from (G). Normalized ratios of LC3-II:LC3-I were quantified. Data are represented as mean ± SD, from 7 independent experiments. WT: wild type, NN: ATG16L1^N206A,N209A^, CC: RAB33B^C227A,C229A^, CC-IF: RAB33B^C227A,C229A,I66A,F95A^, F: Fed, S: Starved, SC: Starved+Chloroquine. n.s.: non-significant, *p ≤ 0.05, **p ≤ 0.01, ***p ≤ 0.001****p ≤ 0.0001 (Student’s t-Test)

In order to investigate the role of RAB33B-ATG16L1 interaction in autophagy, we transfected *atg16l1^−/−^* mouse embryonic fibroblasts (MEFs) [44] with EGFP-WT ATG16L1 and EGFP-ATG16L1^NN^. WT ATG16L1 rescued starvation induced LC3 lipidation and SQSTM1/p62 (sequestosome 1) degradation in *atg16l1^−/−^* MEF, but the rescue was significantly reduced in *atg16l1^−/−^* MEF expressing the RAB33B-binding deficient mutant EGFP-ATG16L1^NN^ (Figure 5). Previous work suggests that overexpression of ATG16L1 or ATG16L1 CCD inhibits autophagy by precluding the ATG12–ATG5-ATG16L1 complex from localizing to the membrane. The authors proposed that a hypothetical ATG16L1-interacting factor is required for membrane localization of the ATG16L1 complex through binding to the CCD of ATG16L1 [45]. To explore the function of RAB33B-ATG16L1 interaction in autophagy, WT ATG16L1 and ATG16L1^NN^, were transiently expressed in EGFP-LC3 stable cell line. Starvation-induced autophagy and autophagic flux were monitored in the presence of chloroquine. Consistent with the previous work, overexpression of the WT ATG16L1 decreased the number of LC3 puncta and LC3 lipidation and blocked SQSTM1 degradation under starvation. However, when ATG16L1^NN^ mutant was overexpressed, LC3 lipidation, autophagosome formation and SQSTM1 degradation remained at similar levels to the control (Figure 6A-D).

Since prenylation is essential for RAB functions on membranes [16,17], RAB33B lacking prenylatable cysteines would exert dominant negative effect as it would not be able to localize on membranes but compete with endogenous RAB33B for binding to ATG16L1. Indeed, expression of RAB33B with prenylatable cysteines C227 and C229 mutated to alanine (RAB33B^CC^) led to inhibition of the formation of EGFP-LC3 puncta, endogenous ATG16L1-structures and LC3 lipidation under starvation, whereas the ATG16L1-binding deficient mutant (RAB33B^CC-IF^) did not (Figure 6E-H). Taken together, these results suggested that the RAB33B-ATG16L1 interaction is important for autophagosome formation.

To investigate the causal relationship between RAB33B and the PtdIns3P-WIPI2B pathway, mCherry-WT RAB33B or its dominant negative mutant, mCherry-RAB33B^CC^, was transfected into human embryonic kidney 293A (HEK293A) cells stably expressing EGFP-WIPI2B, which serves as a sensor for autophagosomal PtdIns3P [9]. WIPI2B puncta formed upon starvation and were sensitive to wortmannin treatment. WT RAB33B- and RAB33B^CC^-expressing cells showed no significant difference in WIPI2B puncta formation (Figure. S5C and S5D). Therefore, the production of PtdIns3P and subsequent recruitment of WIPI2B to membranes were independent of RAB33B.

## Discussion

This is the first report of an endogenous protein that can induce conformational change of a small GTPase from the inactive form to the active form without nucleotide exchange, which is usually the driving force for such a process. It appears that the binding of ATG16L1 with switch I and switch II regions of RAB33B provides energy for such a conformational change in these regions (Figure S3C). Importantly, E186 and R193 in the other molecule of the dimeric ATG16L1 contributed to the stabilization of the active conformation of GDP-RAB33B *via* hydrogen bonding with R94 of the switch II region and the main chain of the switch I region, respectively (Figure 2B).

In a previous affinity-isolation assay, both GTPγS-bound and GDP-bound RAB33B can bind to ATG16L1 with some preference for the GTPγS-bound form [31]. Isothermal titration calorimetry (ITC) measurements show that the affinity of ATG16L1 to GTPγS-RAB33B increases ca. 10-fold in the presence of ATG5. But the affinity of ATG16L1 to GDP-RAB33B remains unchanged in the presence of ATG5 [36]. However, RAB effectors usually bind GTP-RAB with at least three orders of magnitude higher affinity than GDP-RAB [46]. Therefore, ATG16L1 is not considered as a canonical RAB effector. Since both GDP- and GTP-bound RAB33B interact with ATG16L1 with high affinity (K_d_ = 213 nM and 95 nM, respectively) and ATG16L1 can induce conformational change of RAB33B from the inactive to active form, it is conceivable that ATG16L1 may be recruited to the membrane by RAB33B in a nucleotide-independent manner. In other words, the classic GTPase switch may not be required for RAB33B function in autophagosome biogenesis. Such a scenario is not seen in RAB effectors, which preferentially bind to GTP-bound RABs.

e have identified a dyad of invariant hydrophobic residues of RABs, i.e. I66 in the TIGVDF motif in the RABF1 region and F95 in the ERF motif in the RABF3 region, which make conserved hydrophobic interactions with effectors and RBP (Figure S3A and S3E). Mutagenesis analysis suggested that these residues are essential for ATG16L1 binding (Table 1, Figure 3A, 3B and S4). Together with the invariant hydrophobic triad (residues F70, W87 and Y103), the conserved hydrophobic interactions would allow the RAB proteins to recognize various effectors or RBP in order to play different roles during vesicular transport. Furthermore, analysis of RAB-effector structures indicates isoleucine in the TIGVDF motif as a crucial residue for effector binding. Mutation of I66 dramatically decreased the binding affinity with ATG16L1 (Table 1) and disrupted proper localization of RAB33B on the phagophore (Figure 3C-E). Interestingly, K35 from RABSF1 region and Q85 from RABF2 region made hydrogen bond interactions with N206 and N209 of ATG16L1, which are not found in other RAB-effector complexes. These additional bindings were required for the RAB33B-ATG16L1 interaction (Table 1). Disruption of these interactions abrogated the localization of RAB33B and ATG16L1 on the phagophore (Figure 3C-E). Therefore, RAB33B and ATG16L1 mutually determined the localization of each other on the phagophore, which is consistent with our previous finding that effector plays an important role in RAB membrane targeting [38].

The RAB33B-ATG16L1 complex structures showed that the binding sites for RAB33B, WIPI2B and RB1CC1 are adjacent on ATG16L1. WIPI2B binds to the end of C-terminal detached helices of ATG16L1 CCD, located downstream of the RAB33B-bindind site, while the RB1CC1-binding domain resides within the flexible loop between CCD and the WD domain (Figure S1). Using the RAB33B-ATG16L1 complex structure and a model of WIPI2B, we modeled the ternary RAB33B-ATG16L1-WIPI2B complex (Figure S6A and S6B). RAB33B-ATG16L1 interaction was not dependent on WIPI2B binding, since RAB33B bound to ATG16L1 with high affinity in the absence of WIPI2B. However, it has been shown that ATG16L1-WIPI2B interaction is dependent on the CCD of ATG16L1 [9]. We showed that RAB33B pulls down endogenous WIPI2B together with the ATG16L1 complex and dominant negative RAB33B^CC^ inhibits the membrane recruitment of endogenous ATG16L1 (Figures 3B and 6F). These results suggested that RAB33B binding is required for the ATG16L1-WIPI2B interaction on the membrane. Moreover, the high affinity RAB33B-ATG16L1 binding versus the weak ATG16L1-WIPI2B interaction indicates that RAB33B could be the main organizing factor for the RAB33B-ATG16L1-WIPI2B complex. Interestingly, as shown in this study and the previous study [9], both RAB33B-ATG16L1 and WIPI2B-ATG16L1 interactions are required for starvation-induced autophagy. We showed that the PtdIns3P-mediated WIPI2B recruitment to the membrane is independent of RAB33B (Figure S5C and S5D). It is possible that formation of the RAB33B-ATG16L1-WIPI2B ternary complex is required for autophagosome formation. Further work will be required to address how RAB33B and WIPI2B cooperate.

Different regions of ATG16L1 have been shown to be involved in the membrane targeting of ATG16L1 either *via* the direct association with membranes or *via* the binding to proteins under different cellular conditions. The RB1CC1-binding domain (229–242) is required for amino acid starvation-induced autophagy but is indispensable for glucose starvation-induced autophagy [10]. The RB1CC1-binding domain (239–242), W194 and the ubiquitin-binding WD domain of ATG16L1 are required for canonical autophagy and selective autophagy during bacterial infection [12]. We showed that W194 is involved in a key hydrophobic interaction with I66 of RAB33B (Figures 1B and 2B). The WD repeat domain is required for the recruitment of ATG16L1 to single membranes during non-canonical autophagy. Proteins on perturbed endosomal membranes may be involved in the recruitment [14]. The C-terminal region (266–319) of ATG16L1β is involved in direct membrane binding and is dispensable for canonical autophagy but essential for non-canonical autophagy [15]. Three residues of CCD (I171, K179 and R193) are shown to play an important role in the membrane targeting of ATG16L1 during starvation-induced autophagy. However, mutation of these residues also disrupts RAB33B binding [13], probably because R193 of ATG16L1 was involved in the interaction with RAB33B (Figure 2B). Further studies on I171 and K179 (not involved in RAB33B binding) mutants are required to address the function of this region. It is possible that a synergy between the binding of ATG16L1 CCD to lipids and the RAB33B-ATG16L1 interaction may be required for the membrane recruitment of ATG16L1. The weak phenotype of RAB33B knockout and knockdown could be due to the redundancy from RAB33A that can also bind to ATG16L1 [13,31].

Analysis of the dynamics in live cells revealed that RAB33B colocalizes with ATG5- and ATG16L1-positive structures during the entire lifespan of phagophores under starvation, suggesting that RAB33B plays an important role in stabilizing the ATG16L1 complex on the phagophore. Interestingly, RAB33B, ATG16L1 and ATG5 initially assemble on a tubular structure (Figure S5B and Movie S1). It remains elusive where and how these tubular structures originate.

Based on the structures of ATG12–ATG5 in complex with ATG16L1 N terminus (PDB 4GDL) [47], yeast Atg16 CCD (PDB 3A7P) and RAB33B-ATG16L1 complex, we built a model of the RAB33B-(ATG12–ATG5-ATG16L1)-WIPI2B complex on the membrane (Figure S6C). Within ATG16L1, the ATG5 binding domain, RAB33B and WIPI2B binding domain and the WD repeat domain are linked by flexible loops. The 2-fold symmetry of the complex ensures stable association with the membrane, because the entire complex is now anchored to the membrane *via* 4 geranylgeranyl moieties conjugated to the RAB molecules. As a consequence, RAB33B recruits the ATG16L1 complex to the phagophore, thereof may facilitate WIPI2B binding, the conjugation of LC3 to PE and promote autophagosome formation (Figure S6C).

## Materials and methods

### Protein expression and purification

RAB33B-ATG16L1 complexes in both GDP- and GTP-bound form were obtained by protein co-expression. Constitutively active RAB33B^Q92L^ mutant was used to obtain the GTP-bound RAB33B. pOPIN(n)-6xHis-RAB33B (human) and pRSF-ATG16L1 (mouse) were co-transformed into the *Escherichia coli* BL21 (DE3) codon-plus RIL strain (Agilent, 230245). Protein expression was carried out at 20°C with 0.2 mM isopropyl-β-D-1-thiogalactopyranoside (IPTG) (Carl Roth, 2316.4) induction for 16 to 18 h. The cell lysate was subjected to HiTrap (GE Healthcare, 17092104) affinity chromatography and size exclusive chromatography using the buffer: 20 mM 4-(2-hydroxyethyl)-1-piperazineethanesulfonic acid (HEPES) (Carl Roth, 6763.3), pH 7.2, 25 mM NaCl (Carl Roth, P029.2), 2 mM dithioerythritol (DTE) (Sigma, D8255), 2 mM MgCl_2_ (Carl Roth, HN03.1), 10 µM GDP (Sigma Aldrich, G7127) or 10 µM GTP (Sigma Aldrich, G8877). Individual RAB33B and ATG16L1 proteins were cloned into the pOPIN vector with 6xHis tag and GST (glutathione-S-transferase) tag (Gifts from Dortmund Protein Facility), respectively. The proteins were expressed separately and purified using the same procedure. The purified proteins were concentrated and stored at −80°C.

### Nucleotide exchange

RAB33B proteins with a defined nucleotide-binding state were obtained by nucleotide exchange. Ethylenediaminetetraacetic acid (EDTA) (Sigma, E9884) at a final concentration of 5 mM (2.5-fold higher than the concentration of MgCl_2_) was added into the RAB33B protein. Subsequently, 20-fold excess of GppNHp (Jena Bioscience, NU-899), GTP or GDP was added and the solution was incubated at room temperature for 2 h. The protein solution was subjected to buffer exchange [20 mM HEPES (Carl Roth, 6763.3), pH 7.2, 25 mM NaCl (Carl Roth, P029.2), 2 mM DTE (Sigma, D8255), 2 mM MgCl_2_ (Carl Roth, HN03.1) and 10 µM respective nucleotide)] using a NAP5 column (GE Healthcare, 17–0853-01) to remove excess EDTA (Sigma, E9884) and nucleotide. The state of the nucleotide binding was determined by high-performance liquid chromatography using ProntoSIL 120-5-C18-AQ 5 µm (250 x 4.6 mm) column (Bischoff) eluted with buffer [50 mM potassium phosphate (prepared from dipotassium hydrogen phosphate, Sigma, P3786), pH 6.6, 10 mM tetrabutylammonium bromide (Sigma, 193119), 8% acetonitrile (Sigma, 34851)] in a Waters LC system (Waters Corp.).

### Protein crystallization and structural determination

Initial screening was performed with a crystallization reagent kit (QIAGEN and Hampton research), using the sitting drop vapor diffusion technique. The crystals of the GDP-bound RAB33B-ATG16L1 complex were first successfully obtained under a condition containing 20% PEG3350 (Sigma, P4338) and 0.2 M potassium thiocyanate (Sigma, P2713) at 4°C at a concentration of 20 mg/mL. To improve the quality of crystals, the crystallization condition was further optimized in the presence of buffer with various pH ranges using the hanging drop diffusion technique. Improved crystals were yielded in conditions with additional 0.1 M Bis-Tris (Carl Roth, 9140.8) pH 6.5 or 0.1 M sodium potassium phosphate [prepared from disodium hydrogen phosphate (Carl Roth, 4984.1) and potassium dihydrogen phosphate (Carl Roth, 3904.1)] pH 6.5 at 4°C. Single crystals were produced using micro-seeding technique under the identical conditions, yielding crystals diffracting up to 2.4–2.5 Å. Crystals were tested by in-house X-ray diffraction. High-quality datasets were then collected on beamline PX-II of the Swiss Light Source, Switzerland. To prevent radiation damage, the crystals were soaked in reservoir buffer containing 15% glycerol (v/v) as a cryoprotectant. Diffraction data were collected at 100 K.

The GTP-bound RAB33B-ATG16L1 complex was crystallized under identical conditions. Both the GTP- and GDP-bound crystals grew in the P2_1_2_1_2_1_ space group. A molecular replacement method was employed to obtain phase information using the program PHASER [48]. One molecule of GppNHp-bound RAB33B (PDB code 1Z06) and one of the two helices from the coiled-coil structure of BECN1(Beclin 1) (172–257) with the side chains substituted with alanine (PDB code 3Q8T) [49] were used as the search models. The structure was refined with multiple rounds of PHENIX [50] and rebuilt in COOT [51]. Validation was done with MolProbity [52].

The asymmetric unit contained one complex of RAB33B-ATG16L1 with two-fold symmetry. Two ATG16L1 molecules showed a well-defined electron density at the binding interface, which was clearly visible in different electron density maps of the missing side chain of the model. This allowed us to confidentially place the entire missing side chains on the ATG16L1 molecules. The statistics for structural determination and refinement are summarized in Table S1.

### Determination of binding affinity using MST analysis

MST (NanoTemper Technologies GmbH) was employed to determine the mutation effect on the binding affinities of RAB33B and ATG16L1. RAB33B proteins were labeled with Tide Fluor 3 (TF3) maleimide (AAT Bioquest, 2270). Labeled RAB33B was used at a concentration of 10 μM. Unlabeled ATG16L1(141–265) was added in a 1:1 dilution in a concentration from 400 μM to 12 nM. Samples were prepared in a buffer containing 20 mM HEPES (Carl Roth, 6763.3), pH 7.2, 25 mM NaCl (Carl Roth, P029.2), 2 mM DTE (Sigma, D8255), 2 mM MgCl_2_ (Carl Roth, HN03.1), 0.05% Tween 20 (Sigma, P1379) and 10 µM GDP (Sigma, G7127) or GppNHp (Jena Bioscience, NU-899). The samples were filled into standard capillaries and measured with a 38% LED and 60% IR-Laser with laser-on time of 30 s and laser-off time of 5 s. The movement of the molecules along the temperature gradient results in a reduction of fluorescence. Plotting the reduction of fluorescence against the concentration of the titrant ATG16L1(141–265) yielded a non-linear binding curve. The binding curves were fitted with the nonlinear solution of the law of mass action to obtain the dissociation constant.

### In vitro prenylation assay

200 nM RABGGT was incubated with 200 nM CHM and 800 nM {3,7,11-trimethyl-12-(7-nitro-benzo[1,2,5]oxadiazo-4-ylamino),-dodeca-2,6,10-trien-1} pyrophosphate (NBP-FPP) (Jena Bioscience, LI-013) in prenylation buffer containing 20 mM HEPES (Carl Roth, 6763.3), pH 7.2, 25 mM NaCl (Carl Roth, P029.2), 2 mM DTE (Sigma, D8255), 2 mM MgCl_2_ (Carl Roth, HN03.1) for 5 min. The prenylation was initiated by adding 250 nM RAB33B [53]. The fluorescence signal upon the incorporation of {3,7,11-trimethyl-12-(7-nitro-benzo[1,2,5]oxadiazo-4-ylamino),-dodeca-2,6,10-trien-1} pyrophosphate (NBP-FPP) into RAB33B was measured on a Spex Fluoromax-3 spectrofluorometer (Jobin Yvon, Edison). Excitation and emission monochromators were set to 480 nm and 520 nm, respectively. The progress curves were fit to single exponential function using Prism version 7.00 for Windows in order to obtain the half-life of a reaction (t_1/2_).

### Cell culture, antibodies and reagents

Henrietta Lacks (HeLa), HEK293T (ATCC, CRM-CCL-2, CRL-3216,) and HEK293A cells stably expressing EGFP-WIPI2B cells were grown in Minimum Essential Medium Eagle (MEM) (Sigma, M4655) supplemented with 10% fetal bovine serum (FBS) (Invitrogen, 10500), 1% Non-Essential Amino Acids (NEAA) (PAN Biotech GmbH, P08-32,100) and 1% sodium pyruvate (PAN Biotech GmbH, P04-43100). Wild type MCF7 (ATCC, HTB-22) and stably expressing EGFP-LC3, EGFP-ATG16L1 or mCherry-EGFP-LC3 MCF7 cells were grown in MEM supplemented with 10% FBS, 1% NEAA, 1% sodium pyruvate and 10 μg/mL human insulin (Sigma, I9278). Stable MCF7 and HEK293A cells were supplemented with 200 μg/mL G418 (Serva, 49418.03). *atg16l1^−/−^* MEF were grown in Dulbecco’s Modified Eagle’s Medium (DMEM) (Sigma, D5796) supplemented with 10% FBS, 1% NEAA, 1% sodium pyruvate. HEK293A stably expressing EGFP-WIPI2B cells were a kind gift of Dr. Sharon Tooze, The Francis Crick Institute, London, UK. *atg16l1^−/−^* MEF were provided by Prof. Shizuo Akira, Osaka University, Japan. The rest wild type cells were acquired from the ATCC and all cell lines were tested for mycoplasma contamination bimonthly using the MycoAlertTM mycoplasma detection kit (Lonza, LT07-218). HeLa, HEK293T, HEK293A and MCF7 cells were transiently transfected using Xtreme GENE HP DNA transfection reagent (Roche, 06366236001) according to the manufacturer’s instructions. MEF were transiently transfected using Lipofectamine 3000 (Thermo Fisher Scientific, L3000015) according to the manufacturer’s instructions.

The following rabbit antibodies were used: ATG5 (Cell Signaling Technology, 2630), ATG12 (Cell Signaling Technology, 2010), ATG16L1 (Cell Signaling Technology, 8089), GFP (Anaspec, 29779), GOLGA2 (Cell Signaling Technology, 12480), HA (hemagglutinin) tag (Abcam, ab9110), LC3 (Cell Signaling Technology, 2775) and SQSTM1 (MBL International, PM045). Mouse antibodies used were: RAB33B (Frontier Institute Co. Ltd, RAB33bd5-Mo-Tk02), ACTA1/Actin (Chemicon, MAB1501), WIPI2 (AbD Serotec, MCA5780GA), mCherry (Novus Biologicals, NBP1-96,752) and GST (Sigma, SAB4200692). Horseradish peroxidase-conjugated secondary antibodies used for immunoblotting were: anti-mouse (Dako, P0260) and anti-rabbit (Millipore, AQ132P). Secondary antibodies used for immunofluorescence were: anti-rabbit IgG (immunoglobulin G) Alexa Fluor 488 (Jackson ImmunoResearch, 111–545-144), anti-rabbit IgG Alexa Fluor 647 (Jackson ImmunoResearch, 211–605-109) and anti-mouse IgG Alexa Fluor 649 (Jackson ImmunoResearch, 515–495-062).

To manipulate autophagy, the following chemicals were used: 50 μM Chloroquine (BioVision, 1825–100), 50 nM Bafilomycin A1 (BioViotica, BVT-0252-M001), 500 nM Wortmannin (Calbiochem, 681676).

### Cloning and generation of stable cell lines

Human ATG5 was cloned into pHAC2 vector (home made) using XhoI/BamHI restriction sites. Human RAB33B was cloned into pOPIN(n)EGFP, pOPIN(n)Cherry (Dortmund Protein Facility) and pTagBFP-C (Evrogen, FP171) vectors using SalI/BamHI restriction sites. Human ATG16L1 was cloned into pOPIN(n)EGFP, pOPIN(n)Cherry and pOPIN(n)Citrine vectors (Dortmund Protein Facility) or pmTurquoise2 vector (Addgene, 60560, Dorus Gadella lab) using BsrGI/XbaI restriction sites. Mutations were introduced using PCR quick change mutagenesis (sequences of mutagenesis primers used are available on request). Human LC3, ATG5 and ATG16L1 were cloned into pEGFP-C1 (Clontech, 6084–1) vector using BglII/SalI, NheI/BamHI and HindIII/KpnI restriction sites respectively. Resulted EGFP-LC3 was cloned into pmCherry-C1 (Clontech, 362524) vector using XhoI/BamHI restriction sites. pEGFP-C1-LC3, mCherry-pEGFP-C1-LC3, pEGFP-C1-ATG5 and pEGFP-C1-ATG16L1 plasmids were used to generate four human epithelial adenocarcinoma MCF7 stable cell lines. Constructs contain kanamycin resistance for selection in bacteria and neomycin for selection in mammalian cells. To set up stable cell lines, MCF7 cells were transfected with each construct using Xtreme GENE transfection reagent and incubated for 3-4 weeks supplemented with 400 μg/mL G418 (Serva, 49418.03) for selection. Single-cell fluorescence-activated cell sorting was performed (Aria Flow Cytometry System). Stable cell lines derived from single cells homogenously expressed each autophagy-marker.

### Immunoblotting

Cells seeded in 6-well plates were either left untreated or when indicated washed with phosphate-buffered saline (PBS) (Sigma, 806544) and incubated with Earle’s balanced salt solution (EBSS) (Sigma, E2888) for 2 h in the presence or the absence of autophagy modulators as indicated. Then, cells were washed with ice-cold PBS and scraped in 100 μL of RIPA buffer [50 mM Tris-HCl (Sigma, T6066), pH 8, 150 mM NaCl (Sigma, S3014), 0.1% sodium dodecyl sulfate (SDS) (Sigma, L3771), 0.5% sodium deoxycholate (Sigma, D6750), 1% Triton X-100 (Sigma, T8787), 1 mM phenylmethylsulfonyl fluoride (PMSF) (Sigma, P7626), 10 mM sodium fluoride (Sigma, S7920), protease inhibitor cocktail (Roche, 11836145001)]. Lysates were transferred to 1.5 mL microcentrifuge tubes and were centrifuged at 9391 g for 5 min at 4°C. Supernatants were transferred to new tubes and protein concentration was determined via Bradford protein assay (Bio-Rad, 5000001). Equal amounts of total lysate were loaded to sodium dodecyl sulfate-polyacrylamide gel electrophoresis and transferred to nitrocellulose membrane (GE Healthcare, GE10600002). Membranes were blocked with 5% nonfat milk, tris-buffered saline-tween 20 (TBS-T) (Sigma, 91414), washed with TBS-T and incubated with primary antibodies overnight at 4°C. The next day membranes were washed with TBS-T and incubated with secondary antibodies conjugated to horseradish peroxidase for 1 h at room temperature. Finally, membranes were washed with TBS-T, incubated with Amersham enhanced chemiluminescence (GE Healthcare, RPN2124) or SuperSignal West Femto Maximum Sensitivity Substrate (Thermo Fisher Scientific, 34095) for 1 min at room temperature, developed and chemiluminescence was detected using UltraCruz Autoradiography Films (Santa Cruz Biotechnology, sc-201697).

### GST affinity isolation and immunoprecipitation

GST-tagged GDP-bound wild type (GST-WT RAB33B) and ATG16L1-binding deficient mutant (GST-RAB33B^KQ^) RAB33B proteins were loaded on glutathione agarose beads (Thermo Fisher Scientific, 16100). HeLa cells were washed with PBS and incubated with EBSS for 2 h. For WIPI2B affinity isolation, cross linking was performed with 2 mM dithiobis(succinimidyl propionate) (Sigma, D3669) in PBS (Sigma, 806544) for 2 h at 4°C and terminated with 20 mM Tris-HCl (Sigma, T6066), pH 8.0 for 10 min at room temperature. Then, cells were lysed [10 mM Tris-HCl (Sigma, T6066), pH 7.5, 150 mM NaCl (Sigma, S3014), 0.5 mM EDTA (Sigma, E9884), 0.5% NP-40 (Thermo Fisher Scientific, 85124)] and incubated with beads pre-loaded with RAB33B wild type or mutants for 2 h at 4°C. Flow-through was collected and beads were washed with 10-15 column volume of washing buffer [25 mM HEPES (Carl Roth, 6763.3), pH 7.2, 15 mM NaCl (Carl Roth, P029.2) and 2 mM DTE (Sigma, D8255). To elute the conjugated protein from the beads, 1x SDS-loading buffer was added to the beads, boiled for 10 min at 100°C and subjected to immunoblotting analysis.

HEK293T cells were transiently transfected with wild type or indicated mutants of mCherry-tagged RAB33B or ATG16L1 for 48 h. Cells were washed with PBS and incubated with EBSS for 2 h. Then, cells were washed with ice-cold PBS and mCherry-tagged proteins were immunoprecipitated using the RFP-Trap®_MA for immunoprecipitation of RFP (red fluorescent protein)-fusion proteins from mammalian cell extract (Chromotek, rtmak-20) according to the manufacturer’s instructions.

### Immunofluorescence

Cells were transiently transfected and grown on 6-well plates with glass coverslips (Assistant^TM^, Fisher Scientific, 10620962), treated, washed with PBS (Sigma, 806544) and fixed with 4% paraformaldehyde (Thermo Fisher Scientific, 28908) for 15 min before permeabilization with 0.5% Triton-X (Sigma, T8787) for 30 min at room temperature (for anti-HA staining) or 100% methanol (Sigma, 34885-M) for 10 min at −20°C (for anti-ATG16L1, anti-ATG12, anti-GOLGA2 and anti-RAB33B staining). Cells on coverslips were blocked with 5% FBS (Invitrogen, 10500), 0.3% Triton X-100, 0.3 M glycine (Sigma, G8898), PBS for 1 h, washed with PBS and incubated with primary antibody in 1% bovine serum albumin (BSA) (Sigma, A2153), 0.3% Triton X-100, PBS overnight. Then, cells were washed and incubated with secondary antibody in 1% bovine serum albumin (BSA), 0.3% Triton X-100, PBS for 1 h before final washing with PBS and mounting with Aqua-Poly/Mount Coverslipping Medium (Polysciences, 18606).

### Confocal fluorescence microscopy and live cell imaging

HeLa, HEK293A or MCF7 cells were transiently transfected on 35 mm glass bottom dishes (MatTek, P35G-1.5–14-C) for 48 h using X-tremeGENE HP DNA transfection reagent (Roche, 6366546001). Live cell imaging was performed in MEM without phenol red (Thermo Fisher Scientific51200) or EBSS (Sigma, E3024) by using an inverted confocal microscope Leica TCS SP2, SP5 or SP8 AOBS equipped with a 63×/1.4 HCX Plan Apo oil or HC PL APO CS2 63x/1.40 OIL immersion lens and a temperature-controlled hood at 37°C and 5% CO_2_.

TagBFP was exited with 405 nm Cube 1162002/AF laser (Leica SP5). Excitation of EGFP was achieved with the 488 nm argon LGK 7872 ML05 laser (Leica SP2, Leica SP5), 480 white light laser (Leica SP5) or 488 white light laser (Leica SP8). mCherry was exited with 543 nm (Leica SP2), 561/569 nm (Leica SP5), 561 nm DPSS YLK 6120 T02 laser or 595 white light laser (Leica SP8). Alexa Fluor 647 and Dylight 649 were excited using the 633 nm HeNe LGK 7654–15 laser (Leica SP5).

Fluorescence emission was detected using the following band ranges: TagBFP: 420–475 nm (Leica SP5); EGFP: 503–528 nm (Leica SP2), 503–546, 503–560 nm (Leica SP5), 498–560 nm (Leica SP8); mCherry: 600–750 nm or 576–620 nm (when Alexa Fluor 647 was used) (Leica SP5) and 604–750 nm (Leica SP8) and Alexa Fluor 647 or Dylight 649: 648–800 nm (Leica SP5).

Alternatively, live cell imaging was performed using a wide-field microscope Olympus Cell R equipped with a 60×/1.35 UPlanSApo oil objective lens and a temperature-controlled hood at 37°C and 5% CO_2_. mTurquoise2, EGFP and mCherry was excited using BP425-445, BP490-500HQ and BP545-580 excitation filters respectively and detected using BA460-510HQ, BA515-560HQ and BA610IF emission filters respectively.

### FLIM measurements

FLIM experiments and confocal imaging were carried out using the laser scanning confocal microscope FluoView FV1000 (Olympus Deutschland GmbH, Hamburg, Germany) equipped with a time-correlated single-photon counting (TCSPC) LSM Upgrade Kit (PicoQuant).

For confocal images EGFP and Citrine were excited at 488 nm (argon-ion laser) and mCherry at 561 nm [diode-pumped solid-state (DPSS) laser]. The fluorescence signal was detected by an acousto-optic tunable filter (AOTF) and SIM (simultaneous) scanning unit (Olympus Deutschland GmbH). EGFP and Citrine fluorescence was detected through a dichroic mirror (SDM560) at 505–550 and 515–560 nm respectively. mCherry fluorescence was detected at 590–690 nm.

FLIM images were collected through a 60x/1.35 UPlanSApo oil immersion objective (Olympus Deutschland GmbH). For lifetime microscopy of EGFP or Citrine, the samples were excited with a 470 nm pulsed diode laser (LDH 470, PicoQuant, Berlin, Germany) at a repetition rate of 40 MHz. The photons were collected in a single-photon counting avalanche photodiode (PDM Series, MPD, PicoQuant) and timed using a time-correlated single-photon counting module (PicoHarp 300, PicoQuant) after being spectrally filtered using a narrow-band emission filter (HQ 525/15, Chroma, Bellows Falls). All measurements were carried out in an incubation chamber at 37°C and 5% CO_2_.

### FLIM-FRET data and analysis

Lifetime maps were generated using a global analysis MatLab software module [54]. For analysis of the observed lifetime of individual cells and at subcellular resolution, intensity and lifetime images generated by the global analysis script were further processed using ImageJ software [55].

To segment the cells from the background the intensity images where thresholded and a binary mask generated. The background was then removed through multiplying the mask with the lifetime map and the background set to “Not a Number”. The average lifetime of the cell or a specific (cytosolic or punctate) area was then determined using the ROI tool for the respective image section.

Close interaction of fluorescently-tagged ATG16L1 and RAB33B was observed as a decrease in fluorescence lifetime of EGFP/Citrine due to FRET from the donor (EGFP/Citrine) to the acceptor fluorophore (mCherry).

### Correlative light and electron microscopy (CLEM)

HeLa cells were transiently transfected with EGFP-ATG16L1 and mCherry-RAB33B on 35 mm glass bottom dishes with coded square grid (MatTek, P35G-2-14-C-GRID) for 48 h using X-tremeGENE HP DNA transfection reagent (Roche, 6366546001). Then, cells were treated for 2 h, washed with PBS (Sigma, 806544) and fixed with 4% paraformaldehyde (Thermo Fisher Scientific, 28908) for 15 min, washed with PBS and imaged under Leica SP5 confocal microscope equipped with a 63×/1.4 HCX Plan Apo oil immersion lens. Positions of the acquired cells on the coded square grid were stored. Then, cells were washed with PBS, fixed with 2% glutaraldehyde (Sigma, G5882) and 2% sucrose (Sigma, S0389) in PBS (Sigma, P4417) (pH 7.4) for 30 min at room temperature and washed with PBS three times. After post-fixation with 1% osmium tetroxide (OsO_4_) (Sigma, O5500) and 1.5% potassium ferrocyanide K_4_ [Fe(CN)_6_] (Sigma, P9387) for 30 min at 4°C cells were dehydrated in ascending ethanol series incubations and embedded in Epon 812 resin. Ultrathin epon sections were imaged with a FEI Tecnai Spirit 120 kV EM at 4400x nominal magnification. Cells were relocated to electron microscopy with the help of the coded square grid which were stored during light microscopy acquisition. Light microscopy images and respective electron microscopy images were overlaid using TurboReg Plugin of ImageJ software [55].

### Software and statistical analysis

ImageJ software was used to quantify puncta, determine Pearson’s colocalization coefficient from single cells, quantify band intensities and correlate light and electron microscopy images [55]. Statistical analysis was performed using Student’s t-Test as indicated in the figure legends.

## Supplementary Material

Supplemental Material

Supplemental Material
