# Supplementary material for: RAB33B recruits the ATG16L1 complex to the phagophore via a noncanonical RAB binding protein
Source: Autophagy. 2020 Sep 22;17(9):2290–304. doi: 10.1080/15548627.2020.1822629 (PMC8496732; doi:10.1080/15548627.2020.1822629)
Supplement: Supplemental Material [file KAUP_A_1822629_SM6891.docx]

**Supplementary Information**

Supansa Pantoom^a,e,†^, Georgios Konstantinidis^a,f,†^, Stephanie Voss^b,c^, Hongmei Han^c^, Oliver Hofnagel^c^, Zhiyu Li^d,*^, Yao-Wen Wu^a,*^

*^a^Department of Chemistry, Umeå Centre for Microbial Research, Umeå University, SE-90187, Umeå, Sweden; ^b^Chemical Genomics Centre of the Max Planck Society, Otto-Hahn-Str. 15, 44227 Dortmund, Germany; ^c^Max-Planck-Institute of Molecular Physiology, Otto-Hahn-Str. 11, 44227 Dortmund, Germany; ^d^National Cancer Center, National Clinical Research Center for Cancer, Cancer Hospital, Chinese Academy of Medical Sciences and Peking Union Medical College, Beijing, 100021, China; ^e^Translational Neurodegeneration Section “Albrecht-Kossel”, Department of Neurology, University Medical Center Rostock, 18147 Rostock, Germany; ^f^Institute of Molecular Biology and Biotechnology, Foundation for Research and Technology-Hellas, N. Plastira 100, Heraklion 70013, Crete, Greece*

^†^These two authors contributed equally to this work.

^*^Corresponding authors: Yao-Wen Wu, E-mail: [yaowen.wu@umu.se](mailto:yaowen.wu@umu.se); Zhiyu Li, E-mail: lizhiyu2008@hotmail.com

**
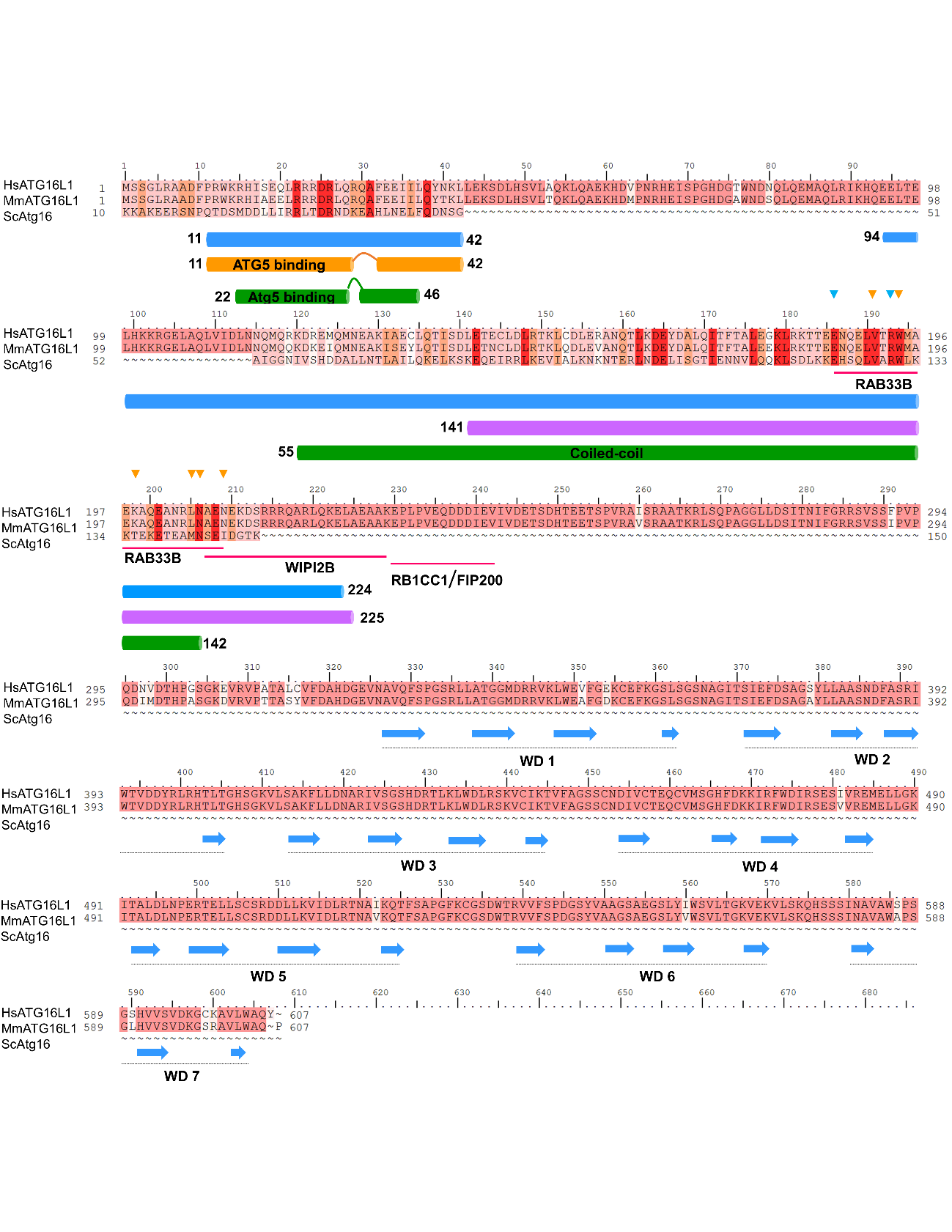
Figure S1.** Sequence alignment of ATG16L1 from different species. Conserved residues in all three species are highlight in red. Amino acid sequences of HsATG16L1 and MsATG16L1 were aligned using multiple sequence viewer (Schrödinger LLC, Mannheim, Germany). The amino acid sequence of ScAtg16 was aligned with HsATG16L1 based on the 3D structure alignment of Atg5 binding domain from yeast Atg16 (PDB 2DYM) and ATG5 binding domain from human ATG16L1 (PDB: 4GDL) as well as the 3D structure alignment of the coiled-coil domain from yeast Atg16 (PDB 3A7P) and the coiled-coil structure observed in this study (PDB 6Y09). Secondary structure derived from 3D structure prediction of MsATG16L1 are shown in blue. The reported 3D structures of the Atg5 binding domain (PDB 2DYM) and the coiled-coil domain of yeast Atg16 (PDB 3A7P) are shown in green. The reported 3D structure for ATG5 binding domain of human ATG16L1 (PDB 4GDL) is shown in orange and the secondary structure of the coiled-coil domain of mouse ATG16L1 derived from this work is shown in violet. Residues involved in interaction with ATG16L1^A^ and ATG16L1^B^ are marked with orange and cyan arrowheads, respectively. Hs: *Homo Sapiens,* Mm: *Mus musculus*, Sc: *Saccharomyces cerevisiae*. Column: α helix, arrow: β sheet.

**
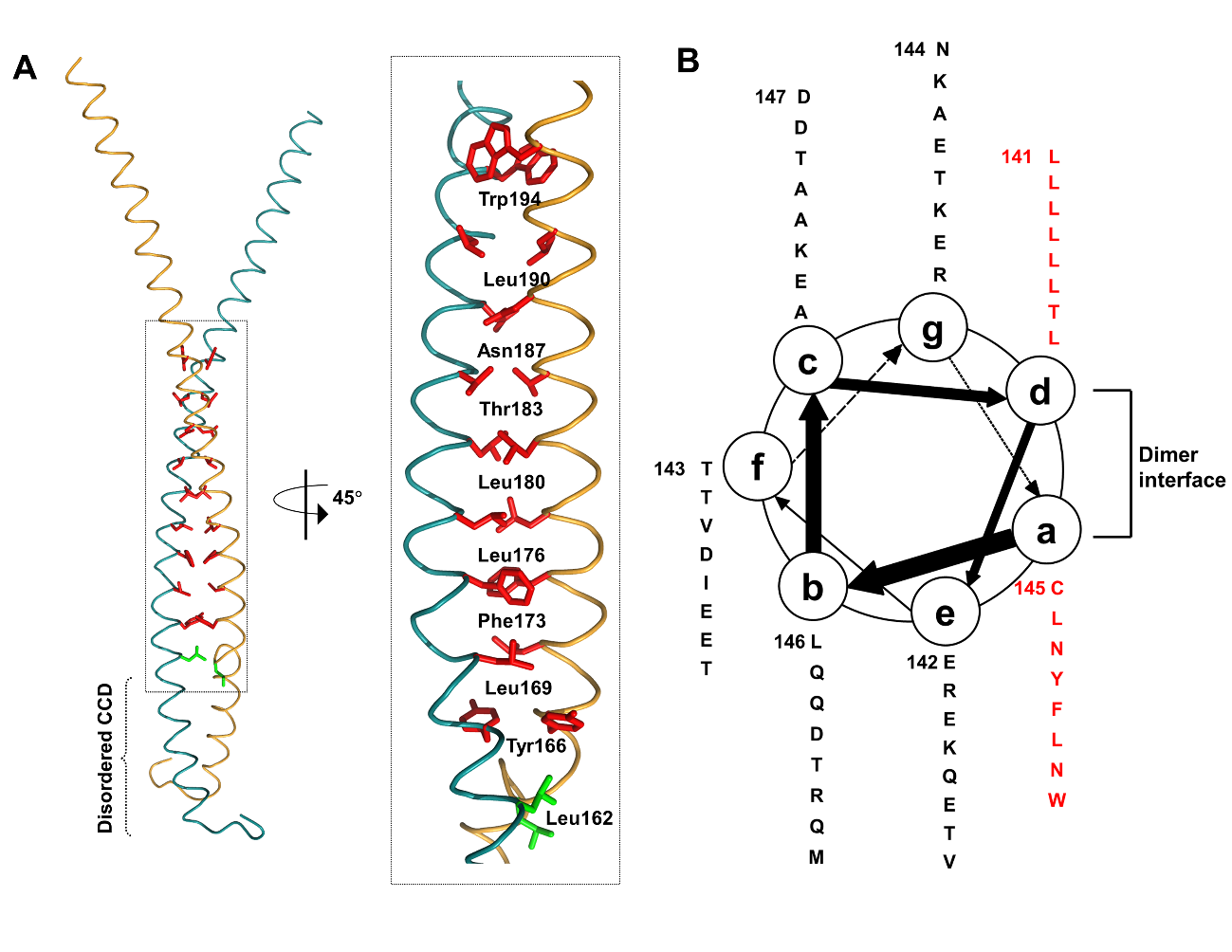
Figure S2.** The dimer interface of ATG16L1 CCD. (**A**) ATG16L1 homodimer is shown as Cα backbone with ATG16L1^A^ and ATG16L1^B^ colored in yellow and cyan, respectively. All the residues at the position *a* and *d* forming *a-a´* and *d-d´* packing are shown as red stick models. The disordered coiled-coil region leads to impaired *a-a´* and *d-d´* packing starting at *d-d´* packing at L162, which is shown as green stick model. (**B**) Helical wheel representation of ATG16L1 CCD. The heptad repeat positions are labeled from a to g. Residues at positions *a* and *d* are colored in red.

**
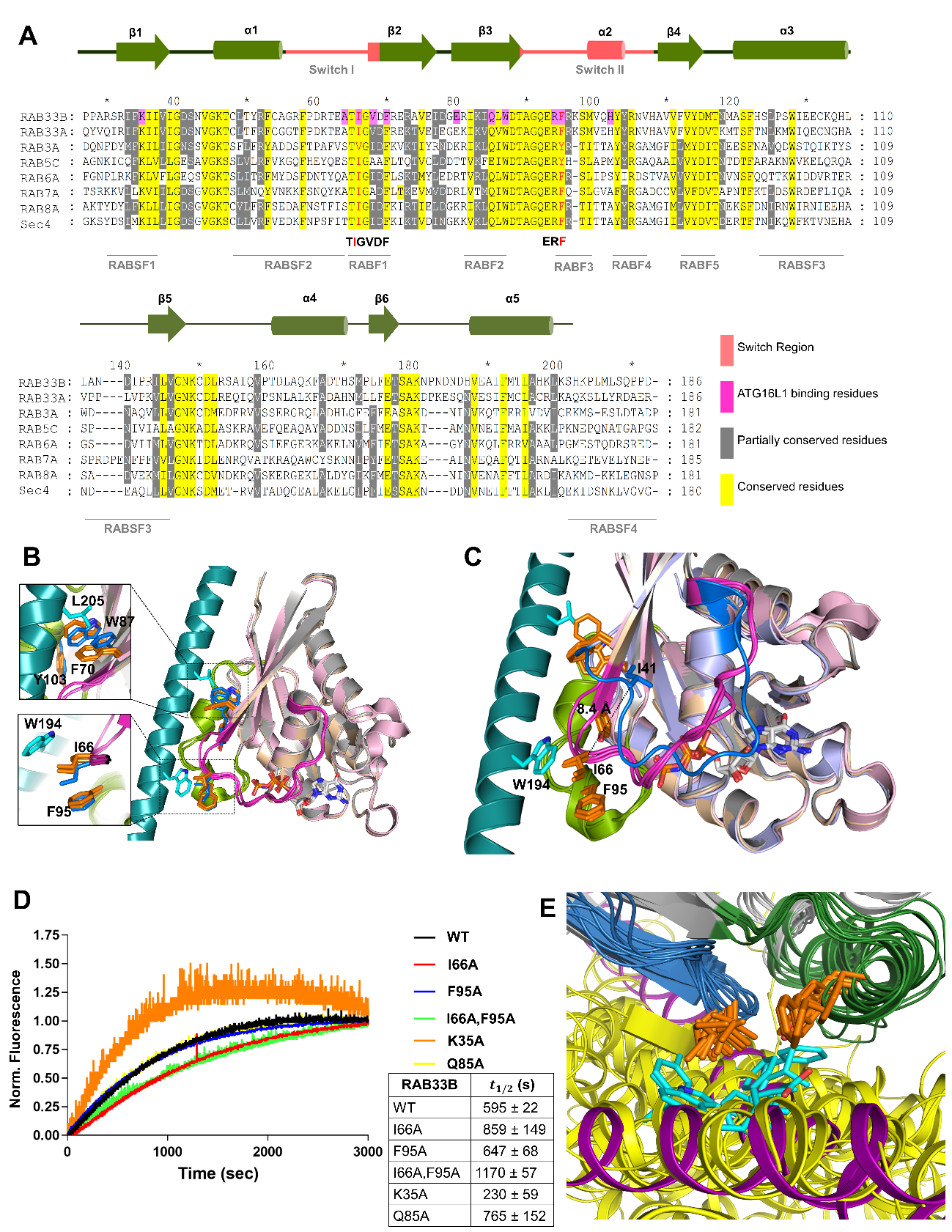
**

**Figure S3.** Structural comparison of free, ATG16L1- and effector-bound-RAB33B in different nucleotide states. (**A**) Sequences of human RAB33B, RAB33A, RAB3A, RAB5C, RAB6A, RAB7A, RAB8A GTPases and yeast Sec4 were aligned using ClustalW2 (http://www.ebi.ac.uk/Tools/msa/clustalw2/). Sequences of RAB GTPases were retrieved from Uniprot Protein Resource (http://www.uniprot.org/). Secondary structure of RAB33B derived from the x-ray structure of the RAB33B-ATG16L1 complex is shown above the aligned sequences, the switch regions are marked in coral. Conserved and partially conserved residues are highlighted in yellow and gray, respectively. The RABSF1-4 and RABF1-5 regions are indicated. Residues involved in interaction with ATG16L1 are labeled in pink. (**B**) Superimposition of free GppNHp-RAB33B (light pink), GTP-bound (gray) and GDP-bound (pale yellow) RAB33B in complex with ATG16L1. Switch I is colored in violet and switch II is colored in green. A helical chain of ATG16L1 involved in RAB33B binding is colored in deep teal. Interacting residues are shown as stick models. The dyad residues I66 and F95 and the triad residues F70, W87 and Y103 of GTP- and GDP-bound RAB33B are colored in orange, while the dyad and triad residues of GppNHp-bound RAB33B are colored in blue. L205 and W194 of ATG16L1 are colored in cyan. (**C**) Superimposition of free GDP-bound RAB1A (light purple) with GTP- (gray) and GDP-bound (pale yellow) RAB33B-ATG16L1 complexes. RAB1A switch I is shown in blue. The rest are colored the same as in (B) (**D**) Effect of RAB33B mutation on prenylation activity. Prenylation time-course of RAB33B and its mutants. Half-life of reactions (t_½_) is shown in the table. Data are represented as mean ± SD, n = 3 measurements. (**E**) Superimposition of RAB protein structures of different RAB-effector complexes (as indicated in the main text) illustrating the conserved hydrophobic dyad residues which mediate the hydrophobic interaction between RAB and respective effector. Dyad residues and the interacting hydrophobic residues on effectors are shown as orange and cyan stick, respectively. ATG16L1 is shown in violet and the other RAB effectors are shown in yellow. Switch I is colored in blue and switch II is colored in green.


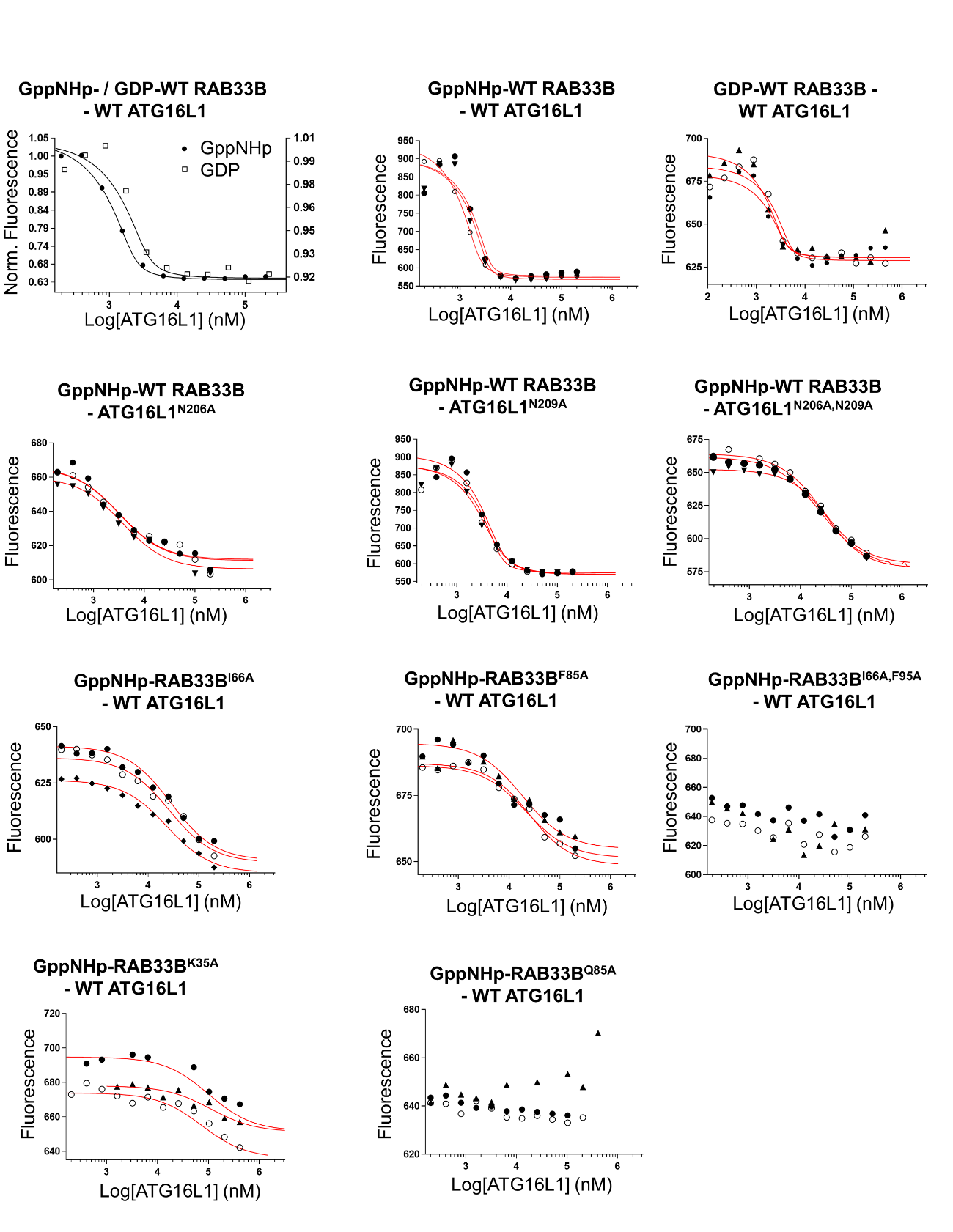


**Figure S4.** MST measurements of binding between RAB33B and ATG16L1 constructs. Change of fluorescence was plotted against log concentration of the titrant ATG16L1 from three independent experiments. The curves were fitted with the nonlinear solution of the law of mass action. Comparison of the binding of GppNHp- and GDP-bound RAB33B with ATG16L1 is shown in the top left panel. Normalized fluorescence of GppNHp- or GDP-bound RAB33B binding to ATG16L1 is plotted on left and right y axis, respectively.

**
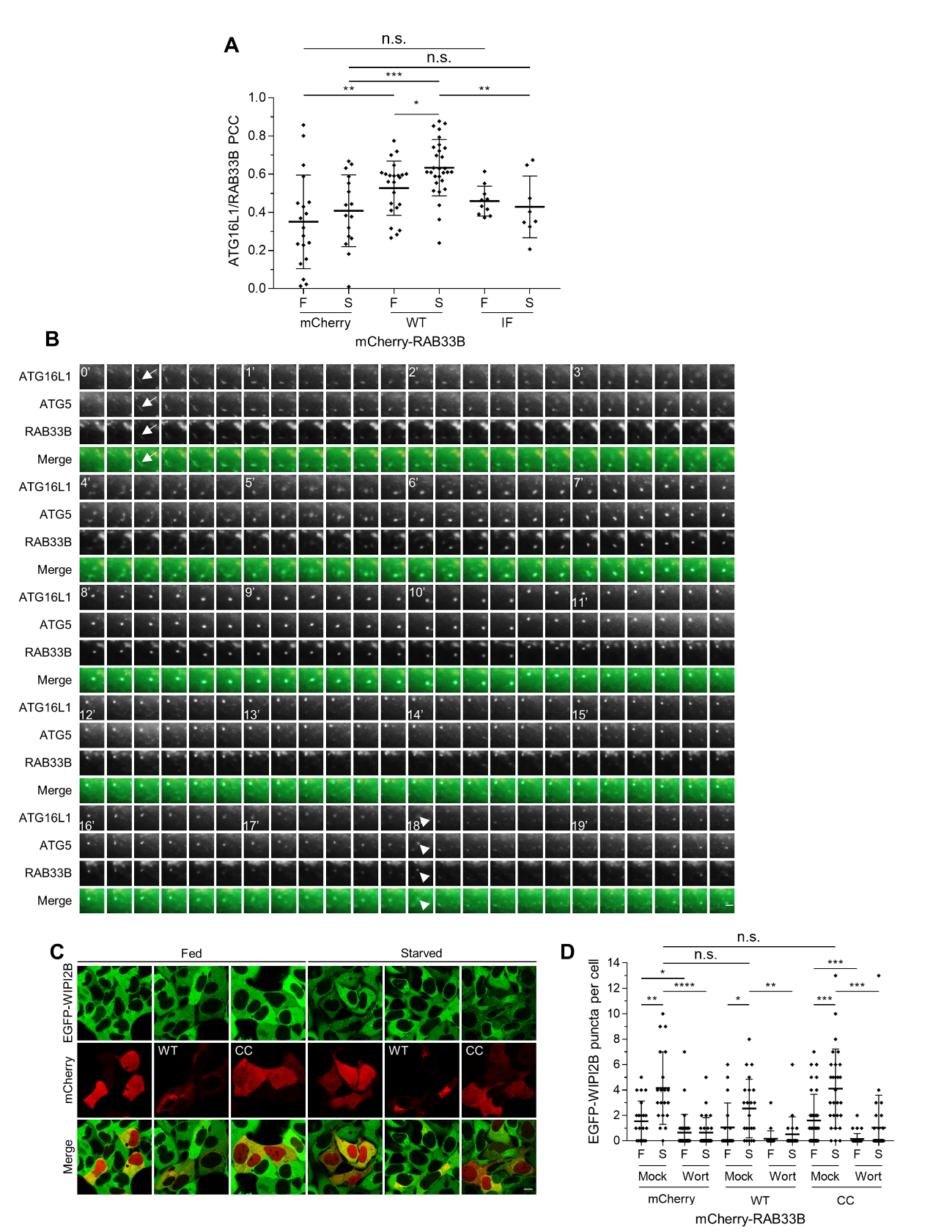
**

**Figure S5.** RAB33B translocates to the phagophore upon starvation and production of PtdIns3P and recruitment of WIPI2B to membranes are independent of RAB33B. (**A**) MCF7 cells stably expressing EGFP-ATG16L1 were transiently transfected with mCherry or the indicated mCherry-RAB33B constructs. Cells were incubated in complete medium (F) or EBSS (S) for 2 h before subjected to confocal microscopy. Pearson’s colocalization coefficient (PCC) was calculated between EGFP-ATG16L1 and mCherry constructs. Data are represented as mean ± SD, n = 8-29 cells. (**B**) HeLa cells transiently expressing mTurquoise2-ATG16L1 WT, EGFP-ATG5 and mCherry-RAB33B WT were cultured for 48 h. Medium was exchanged to EBSS and cells were imaged by wild-field microscopy after 30 min. Montage depicts the lifetime (arrows: appearance, arrowheads: disappearance) of an individual punctate structure positive for ATG16L1, ATG5 and RAB33B (see also Movie S1). Scale bar: 1 μm. (**C**) Stable EGFP-WIPI2B HEK293A cells transiently expressing mCherry or the indicated mCherry-RAB33B constructs were incubated in complete medium (Fed) or EBSS (Starved) for 2 h and imaged by confocal microscopy. Scale bars: 10 μm. (**D**) Quantification of EGFP-WIPI2B puncta per cell from (**C**) in the absence or the presence of 500 nM wortmannin (Wort). Data are represented as mean ± S.D., n = 18-35 cells. WT: RAB33B wild type, IF: RAB33B^I66A,F95A^, CC: RAB33B^C227A,C229A^, F: Fed, S: Starved. n.s.: non-significant, *p ≤ 0.05, **p ≤ 0.01, ***p ≤ 0.001, ****p ≤ 0.0001 (Student’s t-Test).


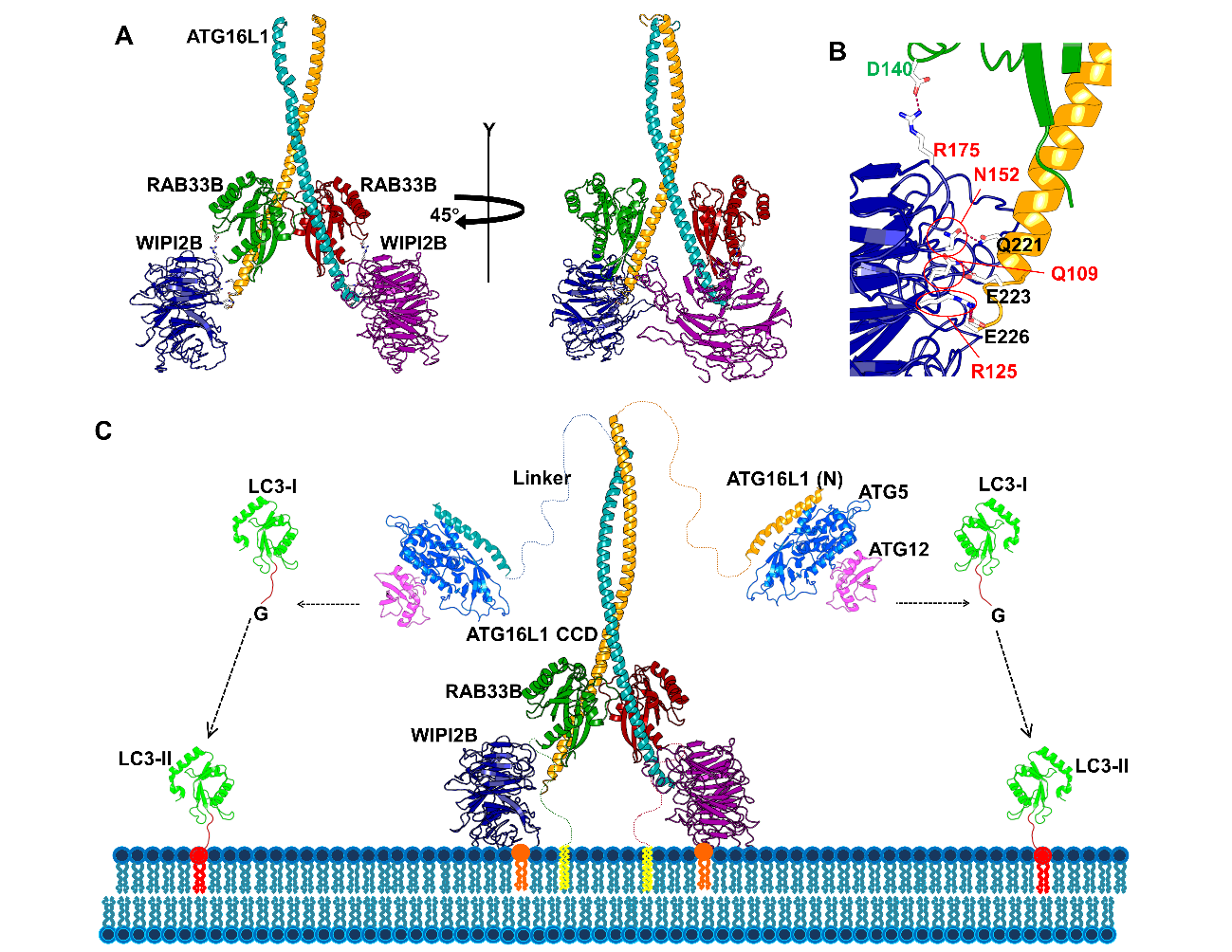


**Figure S6.** Model of the RAB33B-ATG16L1-WIPI2B complex and RAB33B mode of action in autophagy. (**A**) Human WIPI2B was *ab initio* modeled using I-TASSER. The modeled WIPI2B (blue and purple) was docked onto the RAB33B (green and red)-ATG16L1 (cyan and yellow) complex using the automatic protein docking server ZDOCK (http://zdock.umassmed.edu/). Residues R108 and R125 of WIPI2B reported as the key residues involved in interaction with ATG16L1 and residues 220-230 of ATG16L1 reported as the binding site of WIPI2B were constrained to be the binding interface during docking. (**B**) Binding interface of WIPI2B and the RAB33B-ATG16L1 complex. The model shows that residues N152, Q109 and R125 of WIPI2B interact with Q221, E223 and E226 of ATG16L1, respectively. Residue R175 of WIPI2B interacts with D140 of RAB33B. The residues of WIPI2B, ATG16L1 and RAB33B involved in binding are labeled in red, black and green respectively. (**C**) Model of the RAB33B-(ATG12–ATG5-ATG16L1)-WIPI2B complex and its functional role on the phagophore membrane. Lipids in the membrane: PE (red), PtdIns3P (orange), geranylgeranyl (yellow).

**Movie S1.** Lifetime of an individual punctate structure positive for ATG16L1, ATG5 and RAB33B.

**Table S1.** Data collection and refinement statistics.

| **Protein pair** | **RAB33^QL^-ATG16L1** | **WT RAB33-ATG16L1** |
| --- | --- | --- |
| **State** | **GTP-bound** | **GDP-bound** |
| **Data collection** |  |  |
| Space group | P2_1_2_1_2_1_ | P2_1_2_1_2_1_ |
| Cell dimensions |  |  |
| a, b, c (Å) | 56.95, 132.42, 155.13 | 57.01, 132.11, 154.37 |
| α, β, γ (°) | 90, 90, 90 | 90, 90, 90 |
| Wavelength (Å) | 0.97714 | 0.97714 |
| Resolution (Å) | 48.17-2.4 (2.49-2.40)* | 49.57-2.4 (2.49-2.40)* |
| No. total reflections | 602992 (62616) | 609592 (62921) |
| No. unique reflections | 46731 (4618) | 46452 (4577) |
| R_merge_ | 0.139 (1.221) | 0.117 (0.749) |
| R_meas_ | 0.144 (1.262) | 0.122 (0.781) |
| CC_1/2_ | 0.998 (0.871) | 0.999 (0.971) |
| CC* | 1 (0.965) | 1 (0.993) |
| I / σ(I) | 17.0 (2.7) | 16.2 (3.8) |
| Completeness (%) | 99.91 (99.87) | 99.96 (99.89) |
| Redundancy | 12.9 (13.6) | 13.1 (13.7) |
| **Refinement** |  |  |
| No. of reflections used in refinement | 46611 | 46278 |
| No. of reflections used for R_free_ | 2330 | 2317 |
| R_work_ | 19.18 | 18.51 |
| R_free_ | 23.11 | 21.14 |
| No. atoms |  |  |
| Protein | 4258 | 4272 |
| Ligands/ions | 157 | 141 |
| Water | 214 | 179 |
| B-factors (Å^2^**)** |  |  |
| Protein | 64.7 | 70.1 |
| Ligands/ion | 66.8 | 75.9 |
| Water | 53.9 | 56.0 |
| R.m.s deviations |  |  |
| Bond lengths (Å) | 0.008 | 0.009 |
| Bond angles (°) | 1.10 | 1.08 |
| Ramachandran plot (%) |  |  |
| Favored region | 97.28 | 96.71 |
| Allowed region | 2.72 | 3.29 |
| Outlier region | 0 | 0 |
| **PDB entry code** | 6Y09 | 6ZAY |

R_merge_ = Σ_hkl_Σ_i_ | I_i_ (hkl) - <I (hkl)> |/ Σ_hkl_Σ_i_ I_i_ (hkl)

R_meas_ = Σ_hkl_ [N/ (N-1)^1/2^] × Σ_i_ | I_i_(hkl) - <I(hkl)> |/ Σ_hkl_Σ_i_ I_i_(hkl), where I_i_(hkl) is the observed intensity of the ith measurement of an equivalent reflection $(hkl)$ and $N$ is the redundancy.

R-factor = Σhkl | Fo-Fc | /Σ_hkl_ Fo, where F_o_ and F_c_ represent the observed and calculated structure factors, respectively.

R_free_ is obtained for a test set of reflections from 5% of data diffraction.

CC1/2 is the correlation coefficient calculated between two random half data sets.

CC* is CC1/2 modification that demonstrates the correlation of the observed data to unknown true intensities, calculated from CC* = [2 CC_1/2_/ (1+CC_1/2_)]^1/2^.

*Values in parenthesis correspond to the highest-resolution shell.
